# *Entamoeba histolytica* Induce Signaling via Raf/MEK/ERK for Neutrophil Extracellular Trap (NET) Formation

**DOI:** 10.3389/fcimb.2018.00226

**Published:** 2018-07-04

**Authors:** Zayda Fonseca, César Díaz-Godínez, Nancy Mora, Omar R. Alemán, Eileen Uribe-Querol, Julio C. Carrero, Carlos Rosales

**Affiliations:** ^1^Departamento de Inmunología, Instituto de Investigaciones Biomédicas, Universidad Nacional Autónoma de México, Mexico City, Mexico; ^2^División de Estudios de Posgrado e Investigación, Facultad de Odontología, Universidad Nacional Autónoma de México, Mexico City, Mexico

**Keywords:** *Entamoeba histolytica*, neutrophil, NETosis, NETs, ROS, ERK, NF-κB

## Abstract

Amoebiasis, the disease caused by *Entamoeba histolytica* is the third leading cause of human deaths among parasite infections. *E. histolytica* was reported associated with around 100 million cases of amoebic dysentery, colitis and amoebic liver abscess that lead to almost 50,000 fatalities worldwide in 2010. *E. histolytica* infection is associated with the induction of inflammation characterized by a large number of infiltrating neutrophils. These neutrophils have been implicated in defense against this parasite, by mechanisms not completely described. The neutrophil antimicrobial mechanisms include phagocytosis, degranulation, and formation of neutrophil extracellular traps (NETs). Recently, our group reported that NETs are also produced in response to *E. histolytica* trophozoites. But, the mechanism for NETs induction remains unknown. In this report we explored the possibility that *E. histolytica* leads to NETs formation via a signaling pathway similar to the pathways activated by PMA or the Fc receptor FcγRIIIb. Neutrophils were stimulated by *E. histolytica* trophozoites and the effect of various pharmacological inhibitors on amoeba-induced NETs formation was assessed. Selective inhibitors of Raf, MEK, and NF-κB prevented *E. histolytica*-induced NET formation. In contrast, inhibitors of PKC, TAK1, and NADPH-oxidase did not block *E. histolytica*-induced NETs formation. *E. histolytica* induced phosphorylation of ERK in a Raf and MEK dependent manner. These data show that *E. histolytica* activates a signaling pathway to induce NETs formation, that involves Raf/MEK/ERK, but it is independent of PKC, TAK1, and reactive oxygen species (ROS). Thus, amoebas activate neutrophils via a different pathway from the pathways activated by PMA or the IgG receptor FcγRIIIb.

## Introduction

*Entamoeba histolytica* is a protozoan parasite with high prevalence in developing countries (Verkerke and Petri, 2012; Tellevik et al., 2015; Ghenghesh et al., 2016). Amoebiasis, the disease caused by *E. histolytica* affects the intestine and the liver, and is the third leading cause of human deaths among parasite infections (Walsh, 1986; Lozano et al., 2012). In this context, *E. histolytica* was found responsible for about 100 million cases of amoebiasis that led to some 50,000 global deaths in 2010 (Mortimer and Chadee, 2010). Although there is growing understanding of the immune response against amoebas, a full solution to amoebiasis is still needed (Moonah et al., 2013; Nakada-Tsukui and Nozaki, 2016; Cornick and Chadee, 2017). *E. histolytica* infection of the intestine or liver is associated with a strong inflammation characterized by a large number of infiltrating neutrophils (Prathap and Gilman, 1970; Tsutsumi et al., 1984; Tsutsumi and Martinez-Palomo, 1988; Espinosa-Cantellano and Martínez-Palomo, 2000). Usually, large numbers of neutrophil are seen surrounding trophozoites. Yet, amoebas do not seem to be damaged by this interaction. Neutrophils have been implicated in defense against this parasite playing a crucial protective role (Seydel et al., 1997; Velazquez et al., 1998; Jarillo-Luna et al., 2002; Asgharpour et al., 2005; Estrada-Figueroa et al., 2011). However, neutrophils and other leukocytes have also been reported as major inducers of tissue damage during intestinal and liver amoebiasis (Salata and Ravdin, 1986; Pérez-Tamayo et al., 1991, 2006; Seydel et al., 1998; Olivos-García et al., 2007; Dickson-Gonzalez et al., 2009). Therefore, the role of neutrophils in this parasitic infection remains controversial.

Neutrophils, the most abundant leucocytes in peripheral blood, migrate from the circulation to sites of inflammation. Typically, neutrophils are considered the first line of defense because they are the first cells to arrive at the infected site, and they present several antimicrobial functions (Deniset and Kubes, 2014; Mayadas et al., 2014). Among these functions, phagocytosis, degranulation, and formation of neutrophil extracellular traps (NETs) are the most important (Brinkmann et al., 2004; Yipp et al., 2012). NETs are formed by a process known as “NETosis” that involves activation in most cases of nicotinamide adenine dinucleotide phosphate (NADPH)-oxidase, which produces reactive oxygen species (ROS) (Fuchs et al., 2007; Bianchi et al., 2009; Remijsen et al., 2011). NETs are fibers of DNA decorated with histones (Neeli and Radic, 2012) and antimicrobial proteins, such as elastase, myeloperoxidase, lactoferrin, and metalloprotease 9 (Brinkmann et al., 2004; Fuchs et al., 2007). NETs can block the dissemination of microorganisms because they function as a physical barrier where pathogens get caught, and get also exposed to antimicrobial proteins. Consequently, NETs can eliminate pathogens extracellularly and independently of phagocytosis (Papayannopoulos and Zychlinsky, 2009). Several human protozoan parasites have been reported to induce the formation of NETs, including *Leishmania amazonensis, L. major, L. chagasi, Leishmania donovani* (Guimarães-Costa et al., 2009; Gabriel et al., 2010; Hurrell et al., 2015), *Toxoplasma gondii* (Abi Abdallah et al., 2012), and *Trypanosoma cruzi* (Sousa-Rocha et al., 2015). Recently, *E. histolytica* trophozoites were also demonstrated to induce NETs formation (Ávila et al., 2016; Ventura-Juarez et al., 2016). Yet, the mechanism of NETs induction by any of these parasites remains unknown. Although, many microorganisms can induce NETs, no single receptor for pathogen-associated molecular patterns (PAMPs) has been identified as responsible for inducing this neutrophil response. However, Toll-like receptors (TLRs) have been suggested to participate (Yipp et al., 2012). Only two receptors for antibody molecules are reported to be bona fide activators of NETs release from human neutrophils, the IgA receptor FcαR (Aleyd et al., 2014), and the IgG receptor FcγRIIIb (Behnen et al., 2014; Alemán et al., 2016a).

It was firstly published that signaling activated by phorbol 12-myristate 13-acetate (PMA) in neutrophils for NETs formation involves the Raf/ERK pathway (Hakkim et al., 2011) and requires ROS produced by the NADPH-oxidase (Almyroudis et al., 2013). In contrast, we previously found that signaling activated by the FcγRIIIb for NETs formation is different from the pathway activated by PMA (Alemán et al., 2016a,b). For this receptor, NETs formation is dependent on NADPH-oxidase, and extracellular signal-regulated kinase (ERK) activation (Alemán et al., 2016a), and requires signaling through the kinases spleen tyrosine kinase (Syk) and transforming growth factor-β-activated kinase 1 (TAK1) (Alemán et al., 2016b). These results emphasize the recent recognized fact that NETs formation is induced by different signaling pathways depending on diverse stimuli (Kenny et al., 2017). In the case of parasitic pathogens, such as *E. histolytica*, no receptor has been clearly identified as an inducer of NETs, and nothing is known about the signaling pathway activated by amoebas in neutrophils to induce NETs formation. Therefore, in this report we investigated whether *E. histolytica* leads to NETs formation via a signaling pathway that involves ERK activation. Neutrophils were stimulated by *E. histolytica* trophozoites and the effect of various pharmacological inhibitors on amoeba-induced NETs formation was assessed. Selective inhibitors of Raf, MEK, and NF-κB prevented *E. histolytica*-induced NET formation. In contrast, inhibitors of PKC and NADPH-oxidase, as previously reported, blocked PMA-induced (Hakkim et al., 2011), but not *E. histolytica*-induced NET formation. *E. histolytica* induced phosphorylation of ERK in a Raf and MEK dependent manner. Also, NF-κB phosphorylation was dependent on MEK. Our results indicate for the first time that *E. histolytica* triggers a signaling pathway to induce NETs formation, that involves Raf/MEK/ERK, but it is independent of PKC, ROS, Syk, and TAK1. Thus, amoebas activate neutrophils via a different pathway from the pathways activated by PMA or the IgG receptor FcγRIIIb.

## Materials and methods

### Neutrophils

Neutrophils (PMN) were purified from blood exactly as previously described (García-García et al., 2013). Adult healthy volunteers provided a written informed consent before donating blood. The Bioethics Committee at Instituto de Investigaciones Biomédicas—Universidad Nacional Autónoma de México (UNAM), approved the informed consent form, and all experimental procedures.

### Entamoeba histolytica

*Entamoeba histolytica* trophozoites (strain HM1:IMSS) were cultured axenically at 37°C in TYIS-33 medium supplemented with 15% heat-inactivated adult bovine serum and Diamond vitamin Tween® 80 solution (Sigma-Aldrich; St. Louis, MO) (Diamond et al., 1978). The cultures were incubated for 72 h and trophozoites were collected after cooling on ice for 5 min and then centrifuging at 300 × g for 5 min at 4°C. The pelleted trophozoites (amoebas) were resuspended in PBS.

### Reagents

Bovine serum albumin (BSA) was from F. Hoffmann-La Roche Ltd. (Mannheim, Germany). UO126, a specific MEK (ERK kinase) inhibitor was obtained from Promega (Madison, WI, USA). The antibiotic LLZ 1640-2 (also known as (5Z)-7-Oxozeaenol; cas 66018-38-0) (catalog no. sc-202055), a specific TAK1 inhibitor, was from Santa Cruz Biotechnology (Santa Cruz, CA). Wortmannin, a phosphatidylinositol 3-kinase (PI3K) inhibitor; Gö6976, a protein kinase C (PKC) inhibitor; Gö6983, another PKC inhibitor; SB 203580, a p38 MAP kinase inhibitor (catalog number 559389); 4′,6-diamino-2-fenilindol (DAPI), a cell-permeable DNA-binding dye (catalog no. 268298); and 3-(1-methyl-1H-indol-3-yl-methylene)-2-oxo-2,3-dihydro-1H-indole-5-sulfonamide (iSyk), a Syk inhibitor (catalog no. 574711) were from Calbiochem/EMD Millipore (Billerica, MA). The cOmplete™ protease inhibitor cocktail (catalog no. 11697498001) and *PhosSTOP*™ phosphatase inhibitor cocktail (catalog no. 04906845001) were from Roche Diagnostics (Basel, Switzerland). Dihydrorhodamine 123 (catalog no. AS-85711) was from AnaSpec, Inc. (Fremont, CA, USA), and Dihydroethidium (catalog no. 12013) was from Cayman Chemical (Ann Arbor, MI, USA). Diphenyleneiodonium chloride (DPI), an NADPH-oxidase inhibitor (catalog no. D2926); (E)-3-[4-methylphenylsulfonyl]-2-propenenitrile (BAY 117082), an NF-κB inhibitor (catalog no. B5556); 3-(3,5-dibromo-4-hydroxybenzyliden)-5-iodo-1,3-dihydroindol-2-one (GW5074), a cRaf1 kinase inhibitor (catalog no. G6416); phorbol 12-myristate 13-acetate (PMA) (catalog no. P8139), and all other chemicals were from Sigma-Aldrich (St. Louis, MO). The following antibodies were used: rabbit polyclonal anti-histone H4 (acetyl K12) antibody (catalog No. ab61238), and rabbit polyclonal anti-citrulline antibody (catalog No. ab100932) from Abcam, Inc. (Cambridge, MA). Monoclonal antibody IgG2a (IB4) anti-integrin β2 (catalog no. sc-65254), mouse monoclonal IgG2a anti-phospho-ERK 1/2 (pTyr204) (catalog no. sc-7383), mouse monoclonal IgG1 anti-NF-κB p50 subunit (catalog no. sc-8414), and rabbit polyclonal anti phospho-NF-κB p50 subunit (pSer337) (catalog no. sc-33022) from Santa Cruz Biotechnology (Santa Cruz, CA). Monoclonal antibody IgG1 (3G8) anti-human CD16 (catalog no. 556617), and R-phycoerythrin (PE)-conjugated monoclonal antibody IgG2a (clone CLB-gran1 1.5) anti-human CD16b (catalog No. 550868) were from BD Pharmingen™ (San Diego, CA). Tetramethylrhodamine (TRITC) conjugate ZyMax™ goat anti-rabbit IgG (catalog No. 81-6114) was from Thermo Fisher Scientific (Carlsbad, CA). Rabbit monoclonal IgG anti-ERK 1/2 (catalog no. 4695) was from Cell Signaling Technology, Inc. (Beverly, MA). HRP-conjugated F(ab')_2_ goat anti-mouse IgG (catalog No. 0855572), and HRP-conjugated F(ab')_2_ goat anti-rabbit IgG (catalog No. 0855686) were from MP Biomedicals (Santa Ana, CA).

### NET formation assay

Neutrophils (2.5 × 10^5^) in 500 μl RPMI-1640 medium (Gibco®; Grand Island, NY) were added to each well of a 24-well plate (Costar® 3524; Corning Inc., Corning, NY), and incubated in a humidified incubator with 5% CO_2_ at 37°C for 30 min. Then 100 μl of 120 nM PMA in PBS, or 100 μl an *E. histolytica* suspension (1.25 × 10^5^ cell/ml) were added to each well. The amoeba to neutrophil ratio was 1:20, as determined previously (Ávila et al., 2016). Plates were incubated in 5% CO_2_ at 37°C for 4 h. Next, 600 μl of 2% paraformaldehyde in PBS were gently added to each well, and the plates were incubated overnight in 5% CO_2_ at 37°C. The fixative was removed by very gentle aspiration at the side of the well, and then the cells were stained with 150 nM DAPI in PBS for 30 min at room temperature. Finally, the plates were observed with a fluorescence inverted microscope model IX-70 from Olympus (Center Valley, PA). Images were captured with an Evolution-VF Cooled Color camera from Media Cybernetics (Rockville, MD), and the computer program Q Capture pro 6.0 from QIMAGING Surrey (British Columbia, Canada). Images were processed with the computer program ImageJ 1.47v from The National Institutes of Health (Bethesda, MD).

In selected experiments, PMN were incubated on ice for 30 min before stimulation, with the inhibitors: Gö6983 (1 μM), Gö6976 (1 μM), GW5074 (100 μM), UO126 (75 μM), DPI (10 μM), BAY 117082 (5 μM), iSyk (1 μM), antibody IB4 (10 μg/ml), Wortmannin (50 nM), LLZ 1640-2 (10 nM), SB 203580 (200 nM), or the vehicle dimethyl sulfoxide (DMSO) alone.

### Immunofluorescence

For NETs staining, neutrophils (2 × 10^5^) were incubated with *E. histolytica* trophozoites (1 × 10^4^) in 200 μl RPMI-1640 medium (Gibco®; Grand Island, NY) using Lab-Tek^TM^ chamber slides from Thermo Fisher Scientific (Rockford, IL). After 1 h, cultures were fixed with 4% formaldehyde for 10 min, then fixed cells were permeabilized with 0.2% Triton X-100 in PBS for 5 min, and washed three times with PBS. Cells were next blocked with 1% BSA, 0.3 M glycine, 0.1% Tween 20 in PBS for 30 min at 37°C, and then incubated with anti-histone H4 or anti-citrulline antibodies diluted 1/100 in 1% BSA, 0.1% Tween 20 in PBS during 1 h at room temperature. Next, cells were gently washed with cold PBS and incubated in the dark with TRITC–conjugated goat anti-rabbit IgG antibody diluted 1/50 in the same solution for 1 h at room temperature. Cells were finally washed with PBS and stained with 150 nM DAPI. Coverslips were mounted with Fluoroshield before observation in a fluorescence Olympus BX51 microscope.

### Live cell imaging quantification of NETs

NETs formation was quantitated by live imaging and an offline analysis similarly to previous reports (Hoffmann et al., 2016; van der Linden et al., 2017). Neutrophils (2.5 × 10^5^) were incubated in 250 μl RPMI-1640 medium containing 500 nM SYTOX® Green in each well of a 48-well tissue culture plate (Costar® 3548; Corning Inc., Corning, NY). The plate was incubated for 20 min at 37°C in the dark, and then *E. histolytica* trophozoites (1.25 × 10^4^) were added in 50 μl to each well. The amoeba to neutrophil ratio was 1:20. NET release was monitored with an Olympus fluorescence inverted microscope during a period of 240 min. Images were captured every 5 min with an Evolution-VF Cooled Color camera from Media Cybernetics (Rockville, MD), and the computer program Q Capture pro 6.0 from QIMAGING Surrey (British Columbia, Canada). Image files were saved in tiff format and converted to 8-bit grayscale. Images were processed with the computer program Fiji (version 2.0.0-rc-65/1.52b) (Schindelin et al., 2012). This method for NET quantification is based on previously described protocols (Brinkmann et al., 2013; Hoffmann et al., 2016; van der Linden et al., 2017). Briefly, scale was set and threshold adjusted to define the area of fluorescent DNA. Then the area of NETs was measured using the tool “analyze particles.” Total area of all fluorescent particles indicated the amount of NETs formation. Unstimulated neutrophils had an area of 62 ± 6.1 μm^2^. Thus only particles larger than 70 μm^2^ were considered NETs.

### Spectrophotometric quantification of NETs

NET formation was also quantified by detecting DNA release spectrophotometrically with the DNA-binding dye SYTOX® Green as previously described (Behnen et al., 2014; Gonzalez et al., 2014; Alemán et al., 2016a,b). Briefly, neutrophils were resuspended at 1 × 10^6^ cell/ml in RPMI-1640 medium (Gibco®; Grand Island, NY), containing 500 nM SYTOX® Green (Molecular Probes, Inc.; Eugene, OR). Then, 100 μl of this cell suspension (1 × 10^5^ PMN) were added to each well of a 96-well plate (Costar® 3590; Corning Inc., Corning, NY). Next, the plate was incubated at 37°C in a 5% CO_2_ incubator for 20 min. Neutrophils were then stimulated by adding 20 μl of 120 nM PMA (20 nM final concentration), or 20 μl of an *E. histolytica* suspension (2.5 × 10^5^ amoeba/ml) to each corresponding well. The amoeba to neutrophil ratio was 1:20. The plate was then incubated in a 35°C pre-warmed microplate reader, model Synergy HT from BioTek Instruments (Winooski, VT), for up to 4 h. For this assay, cells were not fixed. The fluorescence from the bottom of the plate was read every 5 min, using the 485 nm excitation and 528 emission filters.

### Fluorescent calcium measurements

Neutrophils at 1 × 10^7^ cell/ml in PBS with 1.5 mM Ca^2+^ and 1.5 mM Mg^2+^, were loaded with Fura-2/AM (Calbiochem; San Diego, CA) and cytosolic calcium concentration calculated as previously described (Rosales and Brown, 1991, 1992; García-García et al., 2002). Briefly, 3 × 10^6^ neutrophils in 1 ml PBS were transferred to a cuvette and then 1.5 × 10^5^ amoebas were added in 80 μl PBS. Fluorescence changes were monitored with a Perkin Elmer (Waltham, MA) LS55 spectrofluorimeter, and calcium concentration calculated with the Perkin Elmer FL WinLab software, version 4.00.02.

### FACS

Fluoresce labeling of neutrophil surface receptors for flow cytometry analysis was completed exactly as described (García-García et al., 2007).

### Neutrophil stimulation with trophozoites and protein extraction

Neutrophils (1 × 10^6^) in 500 μl PBS were placed in a 1.5 ml Eppendorf tube. Next, *E. histolytica* trophozoites (5 × 10^4^) in 100 μl PBS were added. Cells were gently mixed and immediately incubated at 37°C in a water bath for various periods of time as indicated. At the end of the corresponding time, 0.8 ml of cold PBS were added and cells centrifuged at 6,000 rpm in a microcentrifuge for 2 min. The supernatant was removed by aspiration and the cell pellets were then lysed in cold RIPA buffer (150 mM NaCl, 5 mM EDTA, 50 mM Hepes, 0.5% sodium deoxycholate, 1% Non-idet P-40, 2 mM Na_3_VO_4_, pH = 7.5), supplemented with 1X cOmplete™ protease inhibitor cocktail and 1X *PhosSTOP*™ phosphatase inhibitor cocktail, which were added just before lysing the cells. Cell lysates were incubated on ice for 20 min, and then cleared by centrifugation. Cell lysates were immediately used for Western blotting.

### Western blotting

Western blots were performed as previously described (Reyes-Reyes et al., 2001). Briefly, proteins in cell lysates were resolved on SDS 10% PAGE, and then electrotransfered onto polyvinylidine fluoride (PVDF) membranes (Immobilon-P; Millipore, Bedford, MA). Membranes were incubated in blocking buffer [1% BSA, 5% non-fat dry milk (Carnation; Nestle, Glendale, CA), and 0.1% Tween® 20 in Tris-buffered saline (TBS: 50 mM Tris-HCl, 150 mM NaCl, pH = 7.5)] for 2 h at room temperature. Membranes were subsequently probed with the corresponding antibody in blocking buffer, overnight at 4°C, anti-phospho-ERK 1 (1/2,500 dilution), or anti-phospho NF-κB (1/2,500 dilution). Membranes were washed with TBS-Tween (TBS containing 0.1% Tween® 20) six times and incubated with a 1/3,000 dilution of the corresponding HRP-conjugated F(ab′)_2_ goat anti-mouse IgG or goat anti-rabbit IgG for 1 h at room temperature. After washing six more times with TBS-Tween, the membrane was developed with Immobilon Western chemiluminescent HRP substrate (catalog no. WBKLS0100) from EMD Millipore (Billerica, MA) according to the manufacturer's instructions. Afterwards, membranes were stripped with 0.2 M NaOH, reprobed with anti-ERK antibody (1/4,000 dilution), or anti-NF-κB antibody (1/2,500 dilution), to assess protein loading in PAGE gels.

### Reactive oxygen species (ROS)

ROS production was assessed with three independent methods (Wojtala et al., 2014). First, the cell-permeative reagent 2′,7′-dichlorofluorescin diacetate (DCFDA) was used in a fluorescence spectroscopy assay. Second, Dihydrorhodamine 123 was used in a flow cytometry (FACS) assay. Third, Dihydroethidium was used in a fluorescence microscopy assay.

For ROS detection with DCFDA, the DCFDA-cellular ROS detection assay kit (catalog no. ab113851) from Abcam, Inc. (Cambridge, MA) was used following the manufacturer's instructions. Briefly, neutrophils were washed with 1X buffer and then incubated with 15 μM DCFDA in 1X buffer for 30 min at 37°C in the dark. After one wash in 1X buffer, neutrophils were resuspended at 1 × 10^6^ cell/ml in 2X buffer. Fifty microliters of this neutrophil suspension (5 × 10^4^ PMN) were added into each well of a 96-well clear-bottom black plate (Costar® 3614; Corning Inc., Corning, NY) and incubated for 20 min at 36°C in a plate-reader, model Synergy HT from BioTek Instruments (Winooski, VT). Then, 50 μl of 40 nM PMA (20 nM final concentration), or 50 μl of an *E. histolytica* suspension (2,500 amoebas) were added. Fluorescence was read every 2 min for 2 h at excitation 485 nm and emission 535 nm.

For ROS detection with dihydrorhodamine 123, neutrophils (1 × 10^6^) were resuspended in 100 μl of 15 μM dihydrorhodamine 123 in PBS inside a 1.5 ml Eppendorf tube, and incubated for 30 min at 37°C in the dark. Neutrophils were then centrifuged at 4,000 rpm for 1 min in a microcentrifuge model 5415C (Eppendorf; Mississauga, Ontario, Canada), and after removing the supernatant, they were washed in 500 μl PBS. Finally, neutrophils were resuspended in 500 μl PBS. In the same tube covered with aluminum foil, 100 μl of 120 nM PMA in PBS (final concentration 20 nM), or 100 μl of an *E. histolytica* suspension (5 × 10^5^ amoebas/ml) were added for PMN stimulation. Cells were incubated for 60 min at 37°C in a 5% CO_2_ incubator, and then fixed by adding 600 μl of 2% paraformaldehyde. Finally, cells were analyzed by flow cytometry using a FACScalibur apparatus (Becton Dickinson; Franklin Lakes, NJ), with the 485 nm (excitation) and 520 nm (emission) filters. PMN were gated by dot-plot analysis and 10,000 cells were acquired per sample. Data analysis was performed using the Cellquest software (Becton Dickinson; Franklin Lakes, NJ).

For ROS detection with dihydroethidium, neutrophils (1 × 10^6^) were resuspended in 100 μl of 15 μM dihydroethidium in PBS, and were incubated for 30 min at 37°C in the dark. Neutrophils were then centrifuged at 4,000 rpm (1,375 × g) for 1 min in a microcentrifuge and washed in 500 μl PBS. Next, neutrophils were resuspended in 500 μl PBS, and 250 μl of this cell suspension (5 × 10^5^ PMN) were added to each well of a 48-well tissue culture plate (Costar® 3548; Corning Inc., Corning, NY). The plate was incubated for 20 min at 37°C in the dark, and then 50 μl of 120 nM de PMA in PBS (20 nM final concentration), or 50 μl of a *E. histolytica* suspension (5 × 10^5^ amoebas/ml) were added to each well. The plate was incubated for 60 min at 37°C in a 5% CO_2_ incubator. Next, 300 μl of 2% paraformaldehyde were added to each well for fixing the cells. After 30 min, the plates were observed with a fluorescence inverted microscope model IX-70 from Olympus (Center Valley, PA).

### Statistical analysis

Quantitative data were expressed as mean ± standard error of mean (SEM). Single variable data were compared by paired-sample Student's *t-*tests using the computer program KaleidaGraph®version 3.6.2 for Mac (Synergy Software; Reading, PA). Differences were considered statistically different at a value *p* < 0.05.

## Results

### *Entamoeba histolytica* induce NET formation

**Figure 1:**
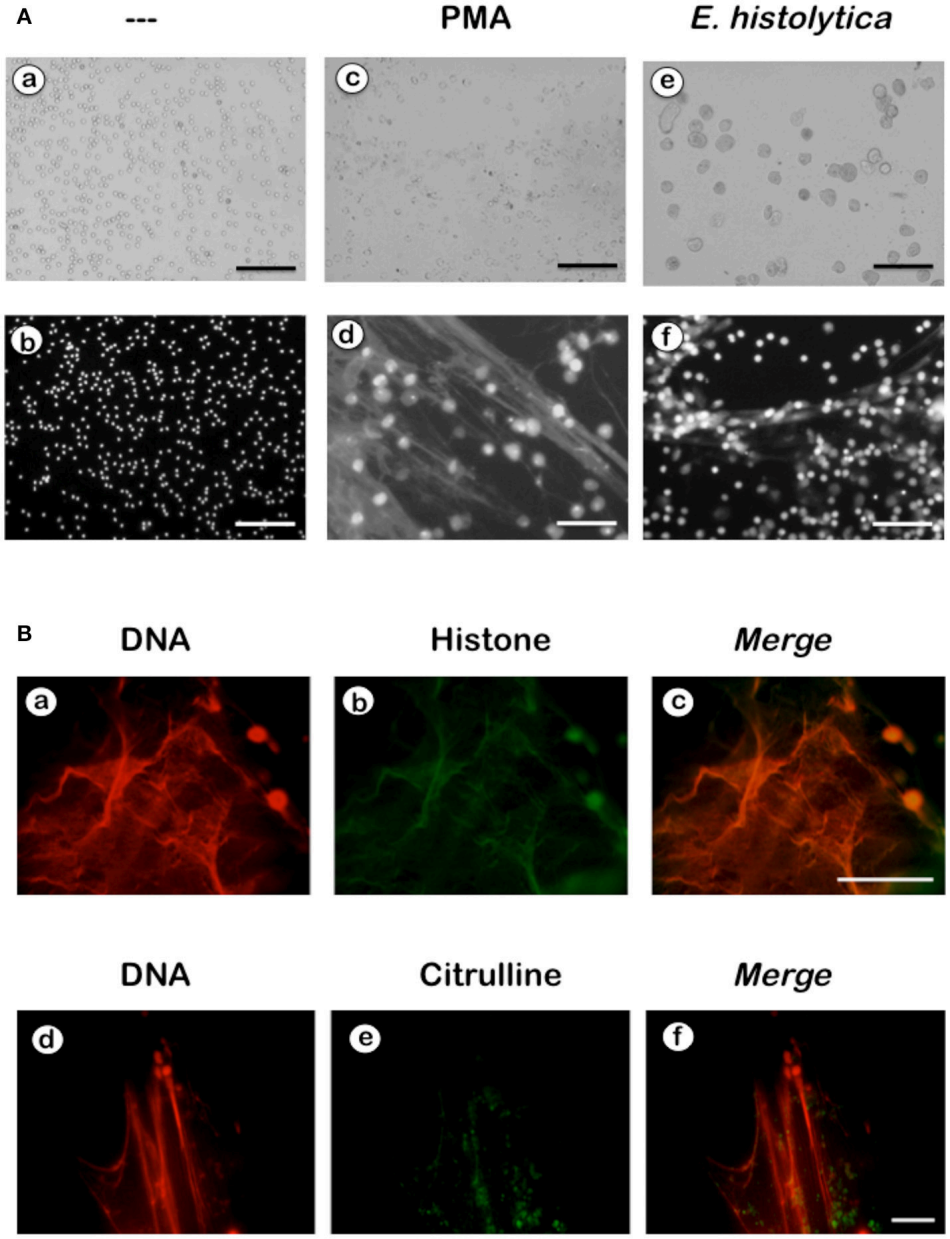
*Entamoeba histolytica* induce NETs formation. **(A)** Human neutrophils (PMN) were left untreated (—), or were stimulated with 20 nM phorbol 12-myristate 13-acetate (PMA), or by *E. histolytica* trophozoites (amoeba to PMN ratio 1:20). After 4 h, cells were fixed and stained for DNA with DAPI. Microphotographs were taken with white light (a,c,e) or with fluorescent light (b,d,f) and are representative of more than 10 experiments. Scale bar is 100 μm. **(B)** Human neutrophils were stimulated with trophozoites during 4 h at 37°C. Cells were fixed and immunofluorescence was performed using anti-histone H4 or anti-citrulline antibodies followed by TRITC-conjugated anti-rabbit IgG antibody. DNA was stained with DAPI. Scale bar 50 is μm.

**Figure 2:**
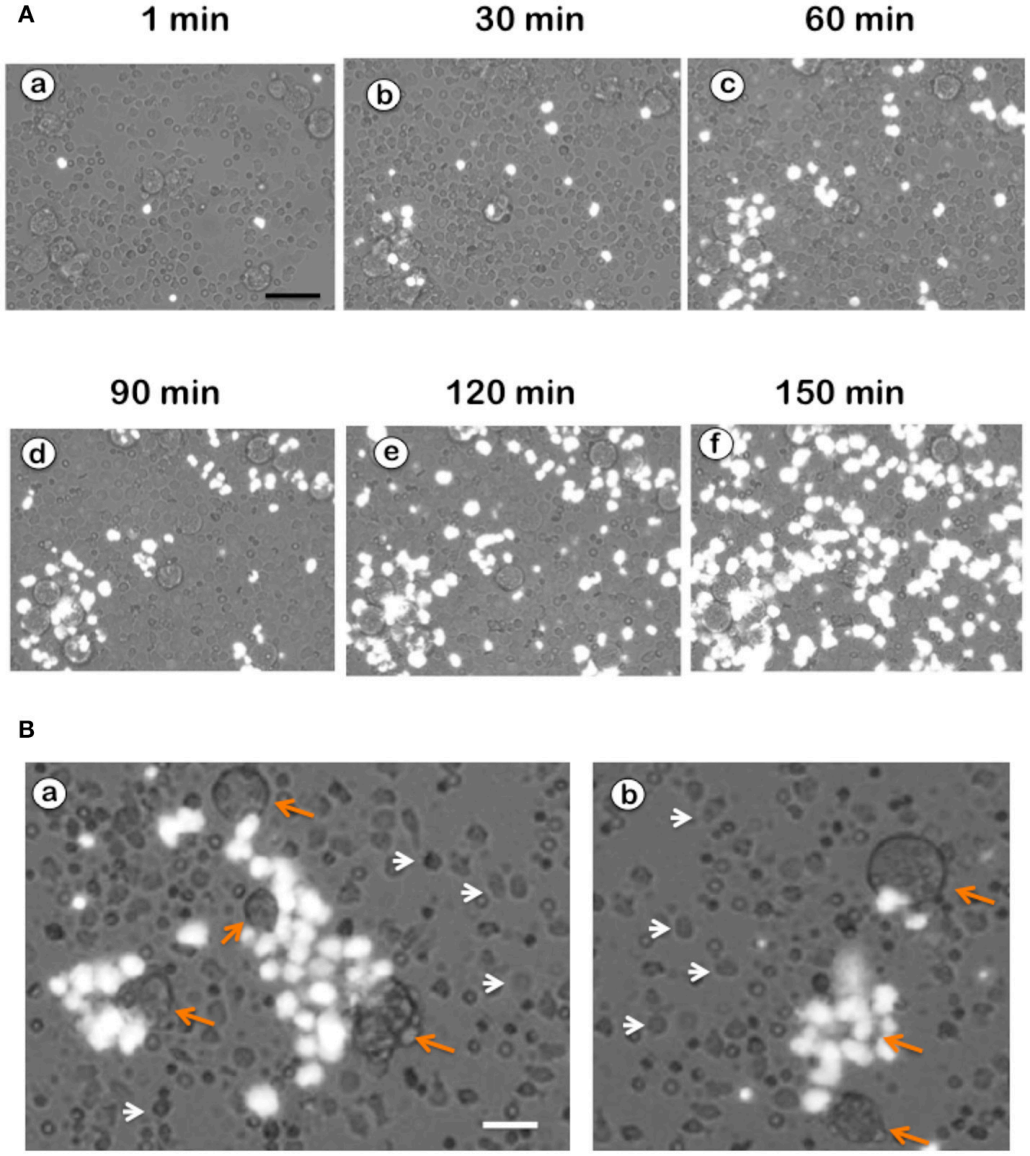
Neutrophils in touch with *Entamoeba histolytica* release NETs. **(A)** Human neutrophils were stimulated with *E. histolytica* in the presence of SYTOX® Green. Live cell images were captured at different times with a fluorescence inverted microscope. External DNA fluorescence appears bright white in the pictures. Scale bar is 50 μm. **(B)** NETs formation was induced only in neutrophils that were in direct contact with *E. histolytica* trophozoites (orange arrows). The NETs were produced around the amoebas and progressively covered the parasites. Neutrophils that were not in contact with amoebas (white arrow heads) did not release DNA fibers and never became SYTOX® Green-positive. Scale bar is 25 μm.

**Figure 3:**
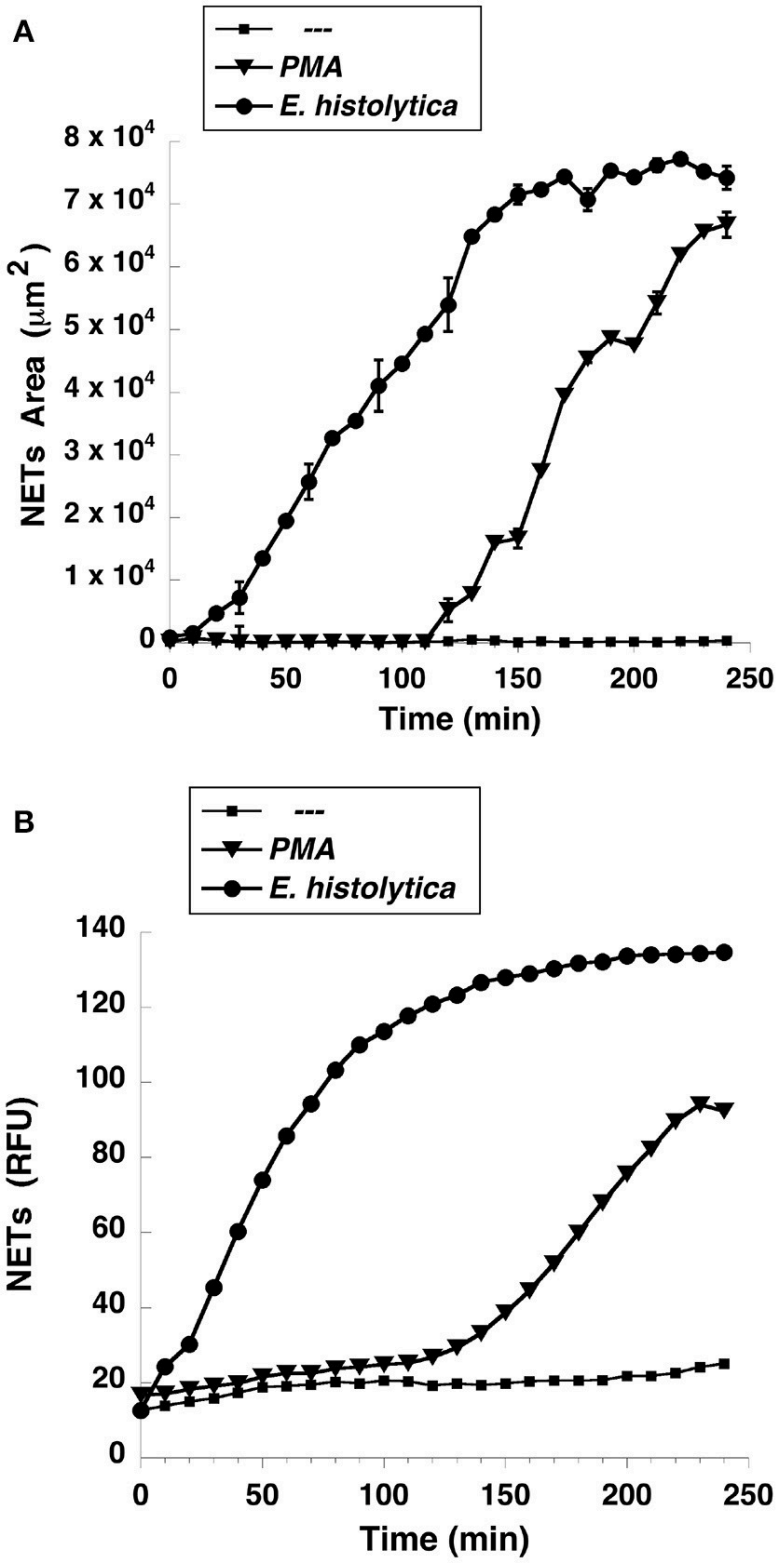
*Entamoeba histolytica* induce NETs formation faster than PMA. Human neutrophils were left untreated (—), or were stimulated with 20 nM phorbol 12-myristate 13-acetate (PMA), or with *E. histolytica*, and then incubated for 4 h. The relative amount of NETs was estimated from **(A)** live cell images and reported as area of external DNA or **(B)** from SYTOX® Green fluorescence in relative fluorescent units (RFU) every 10 min. Data are mean ± SEM of three experiments done in triplicates.

NETs formation has been mostly studied by using PMA, a potent activator of PKC as an inducer of NETosis (Brinkmann et al., 2004). The antibody receptor FcγRIIIb also induces a strong activation of NETs formation (Alemán et al., 2016a,b). In addition, we recently reported that *E. histolytica* also induce NETs formation (Ávila et al., 2016), but there are not reports on the mechanism of NETs induction by these parasites. When human neutrophils were stimulated by PMA, NETs are detected after 2.5 h of stimulation (Fuchs et al., 2007). Complete NETs were seen, as previously reported, by 4 h after stimulation (Figure 1A). Stimulation with *E. histolytica* trophozoites also induced NETosis (Figure 1). The extracellular DNA fibers co-localized with neutrophil elastase and myeloperoxidase (Díaz-Godínez et al., 2018), as well as with histone H4 and citrulline (Figure 1B), confirming that these fibers were bona fide NETs. Live cell imaging showed that by 30 min after incubation with amoebas, NETs were already visible (Figure 2A). By 2 h about half of the total amount of NETs had already been formed, and by 2.5 h NETs reached a maximum level (Figure 2A). NETs formation was induced only in neutrophils that were in direct contact with *E. histolytica* trophozoites (Figure 2B). The NETs were produced around the amoebas and progressively covered the parasites (Figure 2B). Neutrophils that were not in contact with amoebas did not release DNA fibers and never became SYTOX® Green-positive (Figure 2B), suggesting that the signaling for NETosis comes from a receptor that directly recognizes the parasite. Quantification of NETs from live cell imaging analysis showed that amoeba-induced NETs were formed with a much faster kinetics than PMA-induced NETs. After stimulation with amoebas, NETs could be easily detected by 30 min (Figure 3A). The amount of NETs progressively increased during the following 2 h, attaining a maximum level by 2.5 h, that was even higher than the one induced by PMA (Figure 3A). Since NETs were exclusively formed by neutrophils joining amoebas and the fluorescence staining of external DNA only reflected NETs, the formation of NETs was also quantitated spectrophotometrically as previously reported (Alemán et al., 2016a,b). In accordance with live cell imaging analysis, neutrophils treated with PMA showed NETs (extracellular DNA fibers) after 2 h of stimulation reaching a maximum level by 4 h (Figure 3B). Stimulation of neutrophils with *E. histolytica* trophozoites also induced NETosis that could be detected by 30 min and reached a maximum level around 2.5 h (Figure 3B). Neutrophils alone only presented background fluorescence that did not increase during the time of the experiment, thus confirming the imaging data showing that neutrophils did not lose membrane integrity. Therefore, both quantification methods are equivalent and allowed us to reach similar conclusions. The difference in kinetics for NETosis suggested that the signaling induced by *E. histolytica* was different from the one induced by PMA. Hence, we next explored whether the signaling molecules reported to be required for NETs formation after PMA or FcγRIIIb stimulation were also required for *E. histolytica*-induced NETosis.

### *Entamoeba histolytica*-induced NETs formation is dependent on Raf and MEK, but not PKC

**Figure 4:**
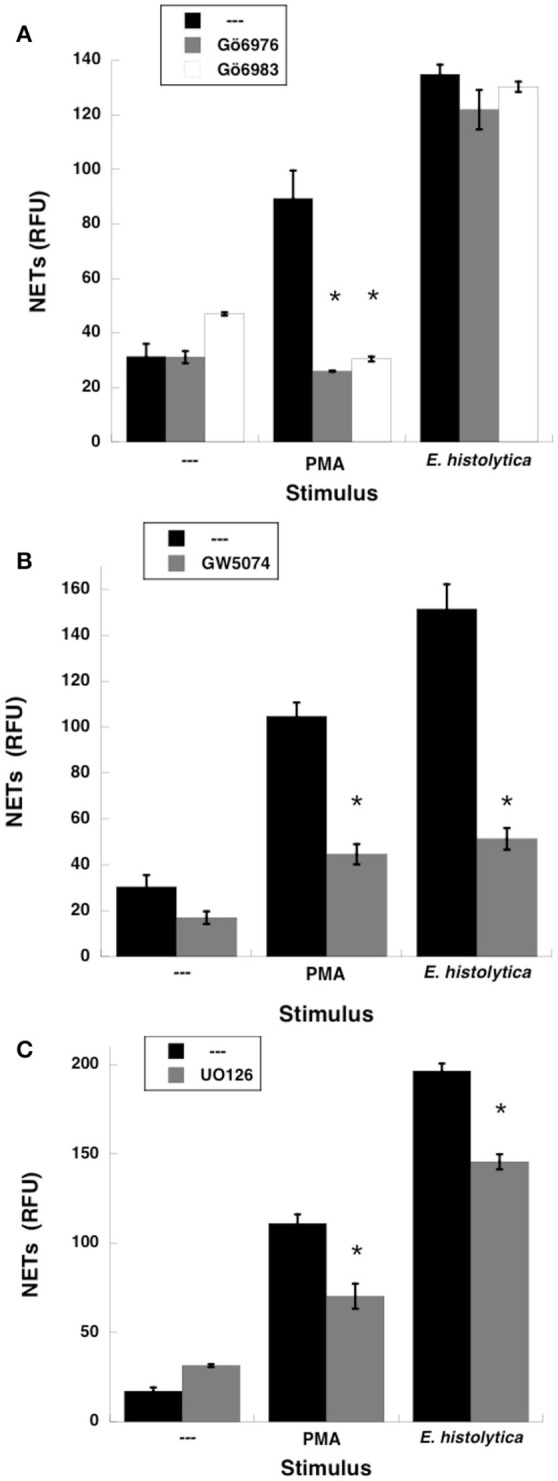
*Entamoeba histolytica-*induced NETs formation is dependent on Raf and MEK, but not *PKC*. Human neutrophils were left untreated (—), stimulated with 20 nM phorbol 12-myristate 13-acetate (PMA), or with *E. histolytica* trophozoites. PMN were previously treated with solvent alone (—) or **(A)** with the PKC inhibitors Gö6976 (1 μM), or Gö6983 (1 μM); or **(B)** with the Raf inhibitor GW5074 (100 μM); or **(C)** with the MEK inhibitor UO126 (75 μM). The relative amount of NETs was estimated from SYTOX® Green fluorescence in relative fluorescent units (RFU) at 4 h after stimulation. Data are mean ± SEM of 3 experiments. Asterisks denote conditions that are statistically different from control (*p* < 0.001).

Because PMA is an activator of PKC, the involvement of this kinase in NET formation induced by *E. histolytica* was tested with two specific PKC inhibitors. PMN treated with PMA formed NETs as expected (Figure 4). However, when PMN were treated previously with Gö6983, an inhibitor of PKCα, PKCβ, and PKCγ isozymes (Figure 4), or with Gö6976, a conventional PKC inhibitor (Figure 4), NETs were not formed after PMA stimulation. In contrast, NETs formation after *E. histolytica* stimulation was not inhibited by these PKC inhibitors (Figure 4, Supplementary Figure 1). In addition, downstream of PKC, the Raf, MEK, ERK pathway has been reported to participate in NETs formation after PMA stimulation (Hakkim et al., 2011). When neutrophils were treated with GW5074, a specific Raf inhibitor, NETs were not formed after PMA stimulation (Figure 4), or after *E. histolytica* stimulation (Figure 4, Supplementary Figure 2). In addition, when PMN were treated with UO126, a potent specific MEK inhibitor, NETs were not formed after PMA stimulation (Figure 4), or after *E. histolytica* stimulation (Figure 4, Supplementary Figure 3). These data suggested that *E. histolytica* stimulation led to NETs formation using Raf and MEK, but not through PKC activation.

### Extracellular calcium is required for *Entamoeba histolytica*-induced NETs formation

**Figure 5:**
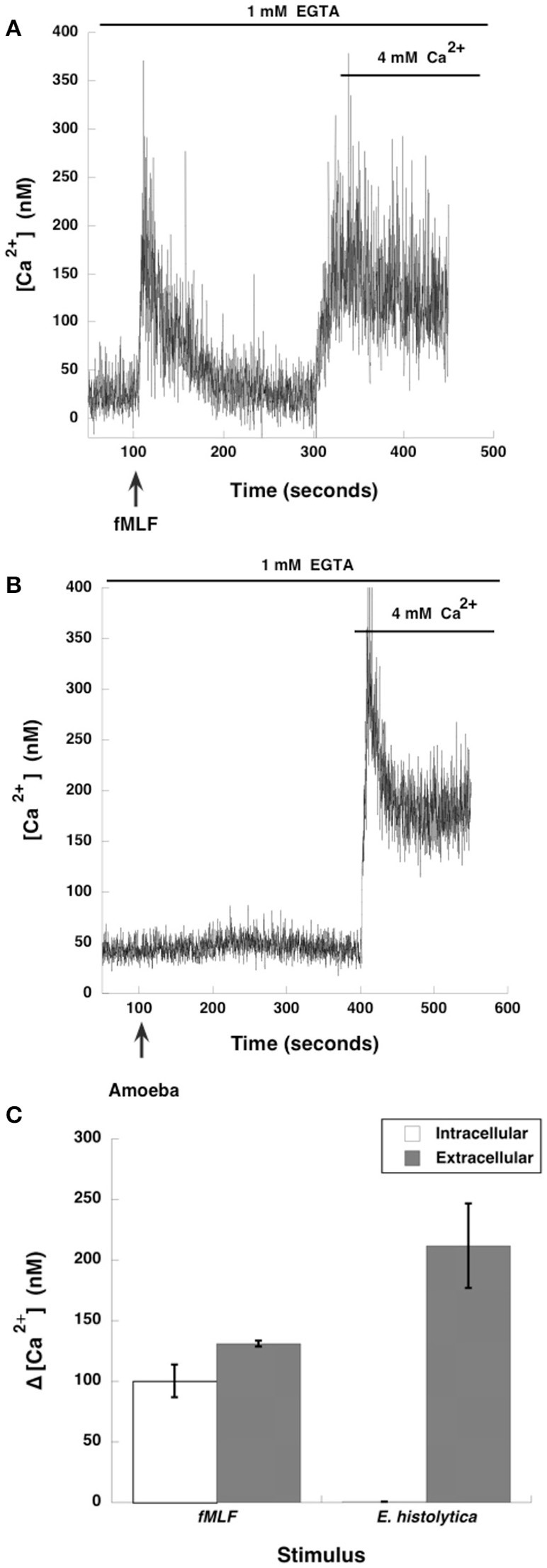
*Entamoeba histolytica* trigger extracellular calcium mobilization in neutrophils. Human neutrophils were incubated with fura-2 for 30 min and then placed in PBS with EGTA. Neutrophils were stimulated (arrow) with **(A)** 10 nM formyl-methionyl-leucyl-phenylalanine (fMLF), or were stimulated with **(B)** *E. histolytica* trophozoites (amoeba). After about 250 s, an excess (4 mM) Ca^2+^ was added to the buffer. Changes in cytosolic calcium concentration were assessed by measuring the variations in fluorescence of fura-2-loaded cells. Tracings are representative of three experiments. **(C)** The increase of cytosolic calcium concentration (Δ [Ca^2+^]) is shown from intracellular or extracellular calcium pools. Data are mean ± SEM of three experiments.

The involvement of MEK in amoeba-induced NETs formation suggested that also ERK would be involved. Since calcium plays a key role in ERK activation, and calcium mobilization is important for NETosis induced by other stimuli (Gupta et al., 2014), we explored the role of intracellular or extracellular calcium pools in *E. histolytica* induced NETosis. Neutrophils were placed in PBS with the calcium chelator EGTA, and then stimulated with the N-formylated chemotactic peptide formyl-methionyl-leucyl-phenylalanine (fMLF). As previously reported (Rosales and Brown, 1992), neutrophils showed an increase in cytosolic calcium concentration that comes from intracellular stores and is dependent on inositol 1,4,5-trisphosphate (IP_3_) (Figure 5A). Then, after adding an excess of calcium outside the cells a second peak of cytosolic calcium was observed, indicating that an extracellular calcium flux is also activated by fMLF (Rosales and Brown, 1991, 1992) (Figure 5A). In contrast, when neutrophils were stimulated with E*. histolytica* trophozoites, no increase in cytosolic calcium concentration was detected. After, addition of an excess of calcium outside the cells, a robust calcium mobilization was observed (Figure 5B). This indicates that amoebas induce a calcium flux in neutrophils that comes only from extracellular pools (Figure 5C). This extracellular calcium flux is important for NETs formation, because NETs were not formed when amoebas interacted with neutrophils in the presence of the calcium chelator EGTA (Díaz-Godínez et al., 2018). It has also been suggested that calcium mobilization required for NETosis involves the calcineurin pathway since cyclosporine A inhibited IL-8-induced NETosis (Gupta et al., 2014). However, in the case of amoebas, cyclosporine A did not prevent calcium mobilization nor NETs formation (data not shown). This suggests that *E. histolytica-*induced NETs formation requires extracellular calcium, but this calcium does not activate the calcineurin pathway.

### *Entamoeba histolytica* trophozoites induced NETosis but not apoptosis

**Figure 6:**
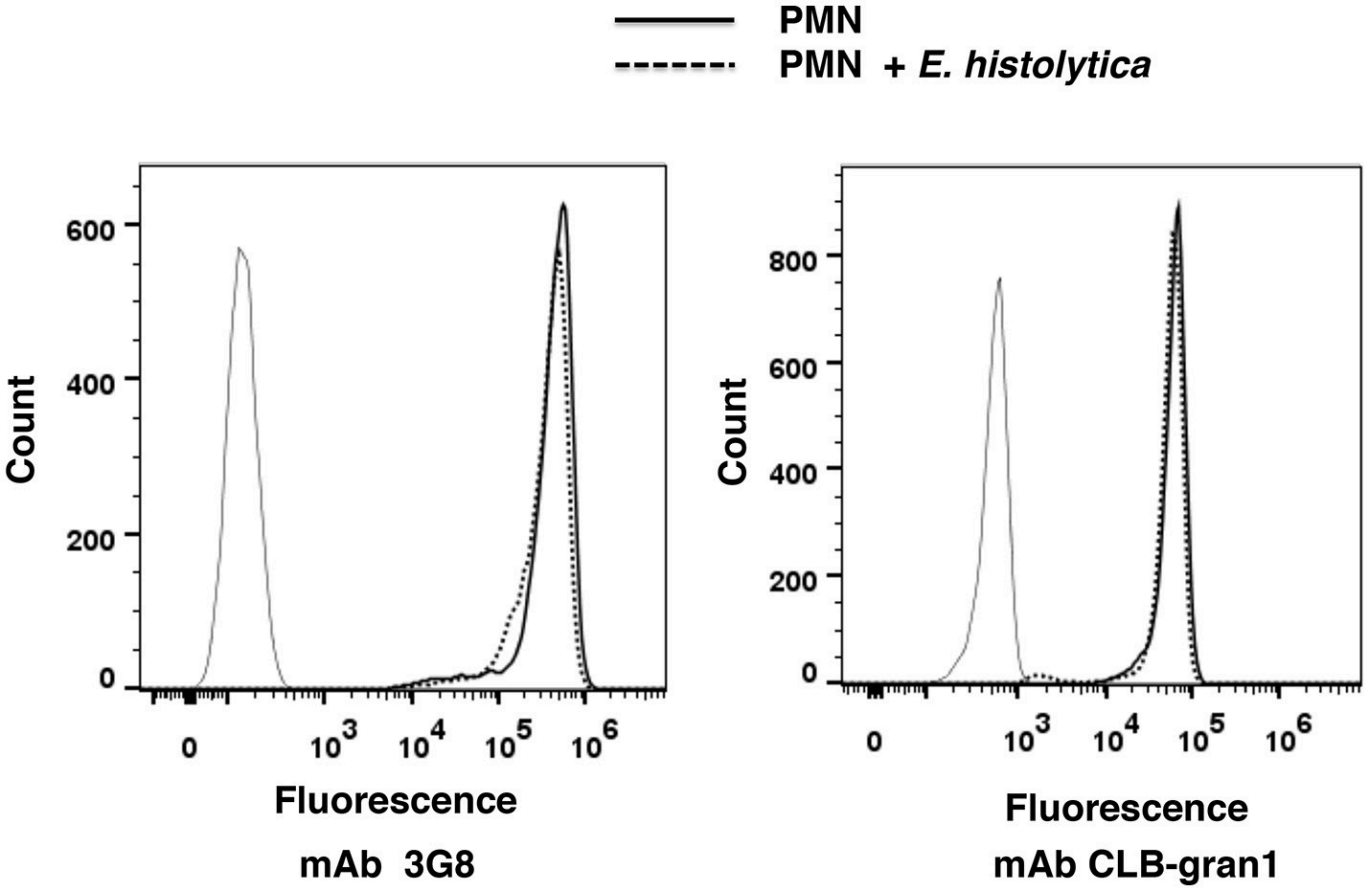
*Entamoeba histolytica* trophozoites did not change surface expression of receptor FcγRIIIb (CD16b). Human neutrophils were not stimulated (solid line) or were stimulated with *E. histolytica* trophozoites (dashed line) for 30 min at 37°C. Then, cells were fluorescence-stained with secondary antibody only (thin line), or with monoclonal antibody (mAb) 3G8 or with mAb CLB-gran1, both specific against FcγRIIIb, as described in section Materials and Methods. Fluorescence intensity was then analyzed by flow cytometry. Histograms are representative of three independent determinations.

Live cell imaging revealed that only neutrophils in contact with amoebas released their DNA (Figure 2). In order to confirm that neutrophils were undergoing NETosis and no other forms of cell death in the presence of amoebas, the integrity of chromatin was analyzed. DNA from neutrophils treated with PMA, or with the calcium ionophore A23187, or exposed to amoebas did not show any fragmentation in agarose gels (Díaz-Godínez et al., 2018). In contrast, neutrophils exposed to 56°C for 1 h, a well-known inducer of apoptosis, showed fragmented DNA (Díaz-Godínez et al., 2018). Moreover, heat-treated neutrophils, and some PMA-treated neutrophils, showed an increase in surface expression of phosphatidylserine; while neutrophils exposed to A23187 or to amoebas did not show phosphatidylserine surface expression (Díaz-Godínez et al., 2018). In addition, it has been reported that neutrophils undergoing apoptosis lose the expression of the antibody receptor FcγRIIIb (CD16b) (Sim et al., 2005). However, neutrophils exposed to *E. histolytica* trophozoites, did not show any difference in FcγRIIIb expression, as indicated by binding of two different monoclonal antibodies specific for this receptor (Figure 6). Together these results confirm that amoebas induce NETs formation and not apoptosis when they are in contact with neutrophils.

### *Entamoeba histolytica*-induced NETs formation is dependent on ERK

**Figure 7:**
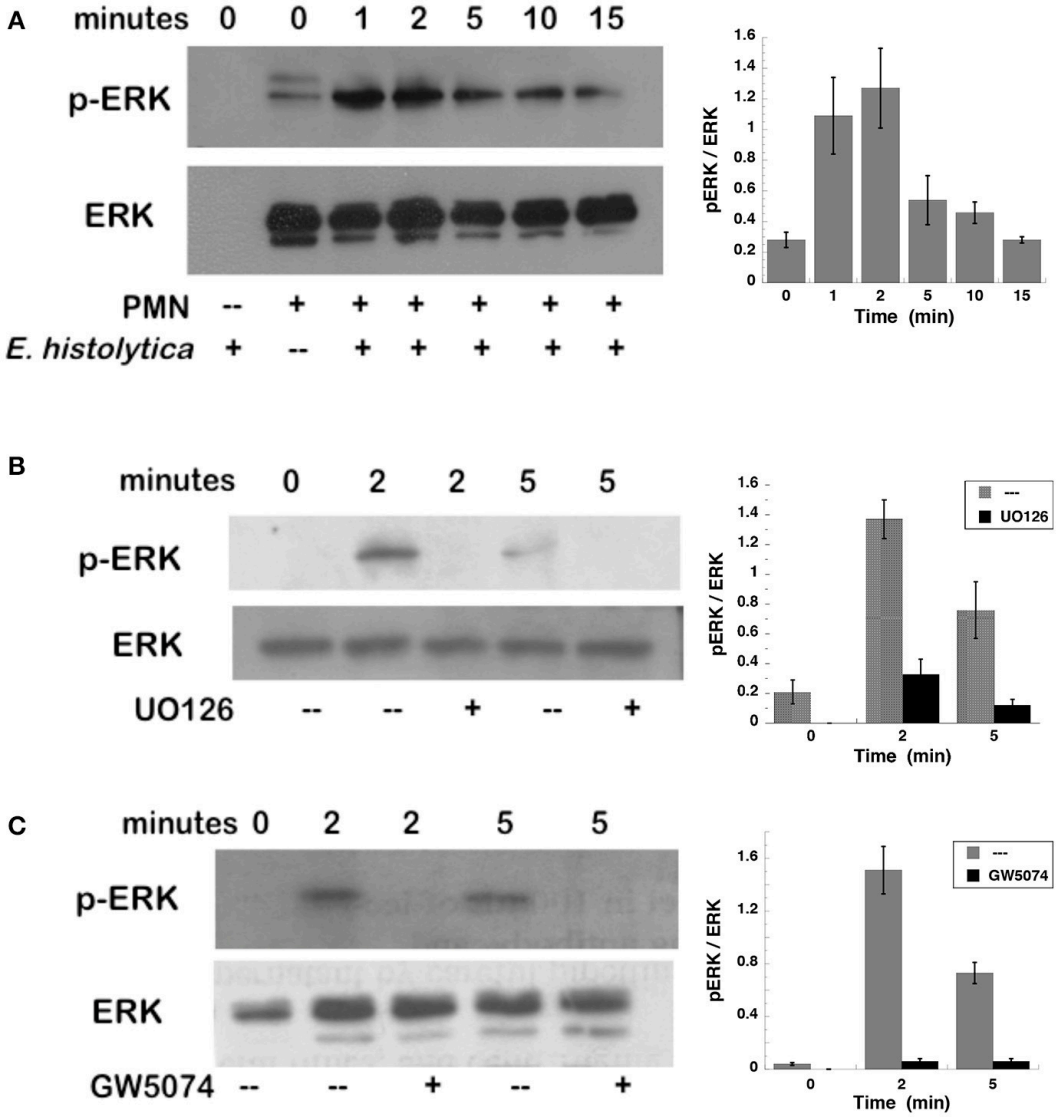
*Entamoeba histolytica* induce activation of ERK. Human neutrophils were stimulated with *E. histolytica* trophozoites for various periods of time, and then cell lysates were prepared. Neutrophils were **(A)** left untreated, or previously treated with **(B)** the MEK inhibitor UO126 (75 μM), or with **(C)** the Raf inhibitor GW5074 (100 μM). Proteins were resolved by SDS-PAGE, and then Western blotted for phosphorylated-ERK (p-ERK) (upper panel) or for total ERK (lower panel) to show equal loading of proteins. Plots on the right show densitometric analysis for the ratio of pERK/ERK. Data are mean ± SEM of three experiments.

To confirm that ERK was activated after *E. histolytica* stimulation, neutrophils with or without the MEK inhibitor were incubated with amoebas and then ERK activation was detected by Western blotting. *E. histolytica* induced a rapid ERK phosphorylation, which reached a maximum at about 2 min after neutrophils and amoebas got in contact (Figure 7A). This phosphorylation then decreased with time and was barely detectable after 15 min. The antibodies used to detect ERK and phospho-ERK did not recognize any proteins from amoeba cell lysates (Figure 7A), thus ERK phosphorylation detected was only from neutrophils. *E. histolytica*-induced ERK phosphorylation was prevented by the MEK inhibitor UO126 (Figure 7B). Also, the Raf inhibitor GW5074 completely impeded *E. histolytica*-induced ERK phosphorylation (Figure 7C). These data suggested that *E. histolytica* induced, in neutrophils, the Raf, MEK, ERK signaling pathway to activate NETosis.

### The NADPH-oxidase inhibitor DPI reduced *Entamoeba histolytica*-induced NETs formation

**Figure 8:**
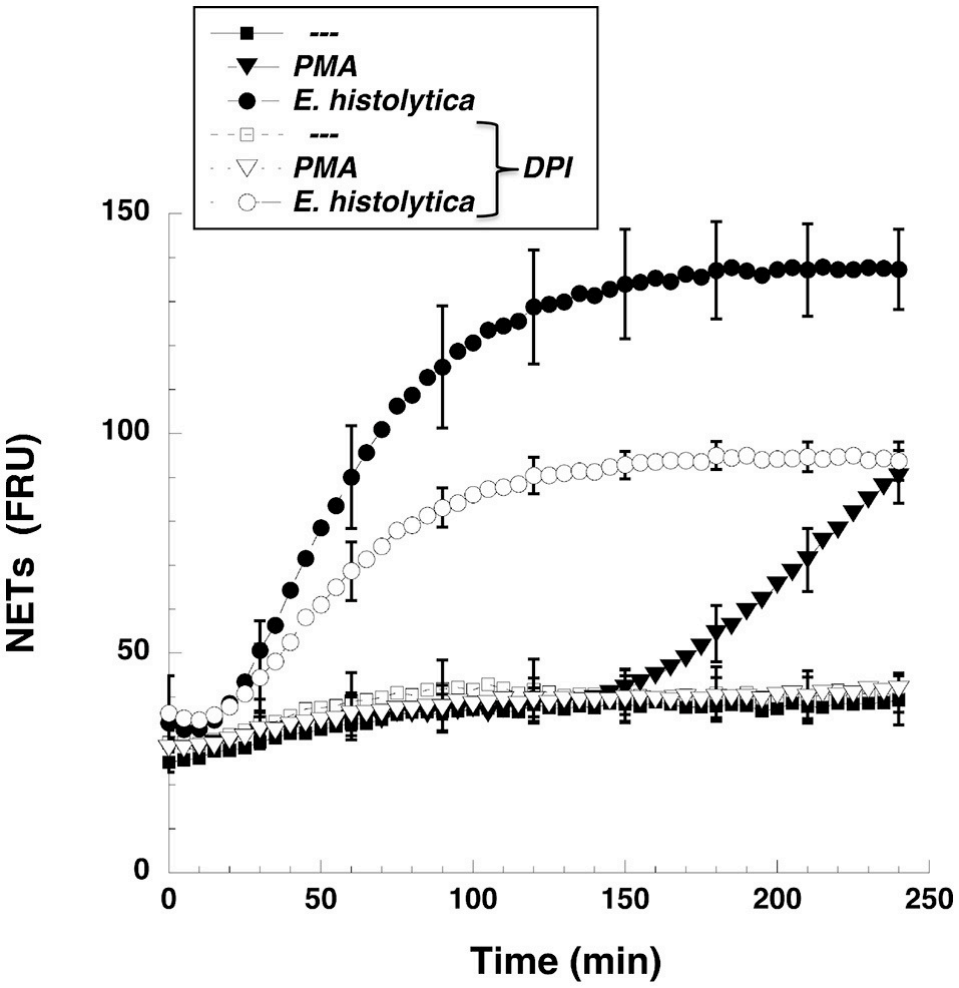
The NADPH-oxidase inhibitor DPI reduced *Entamoeba histolytica-*induced NETs formation. Human neutrophils were not stimulated (—), or were stimulated with 20 nM phorbol 12-myristate 13-acetate (PMA), or with *E. histolytica trophozoites*. Some neutrophils were previously treated with 10 μM diphenyleneiodonium (DPI), a NADPH-oxidase inhibitor (open symbols). The relative amount of NETs was estimated from SYTOX® Green fluorescence in relative fluorescent units (RFU) during 4 h after stimulation. Data are mean ± SEM of three experiments.

**Figure 9:**
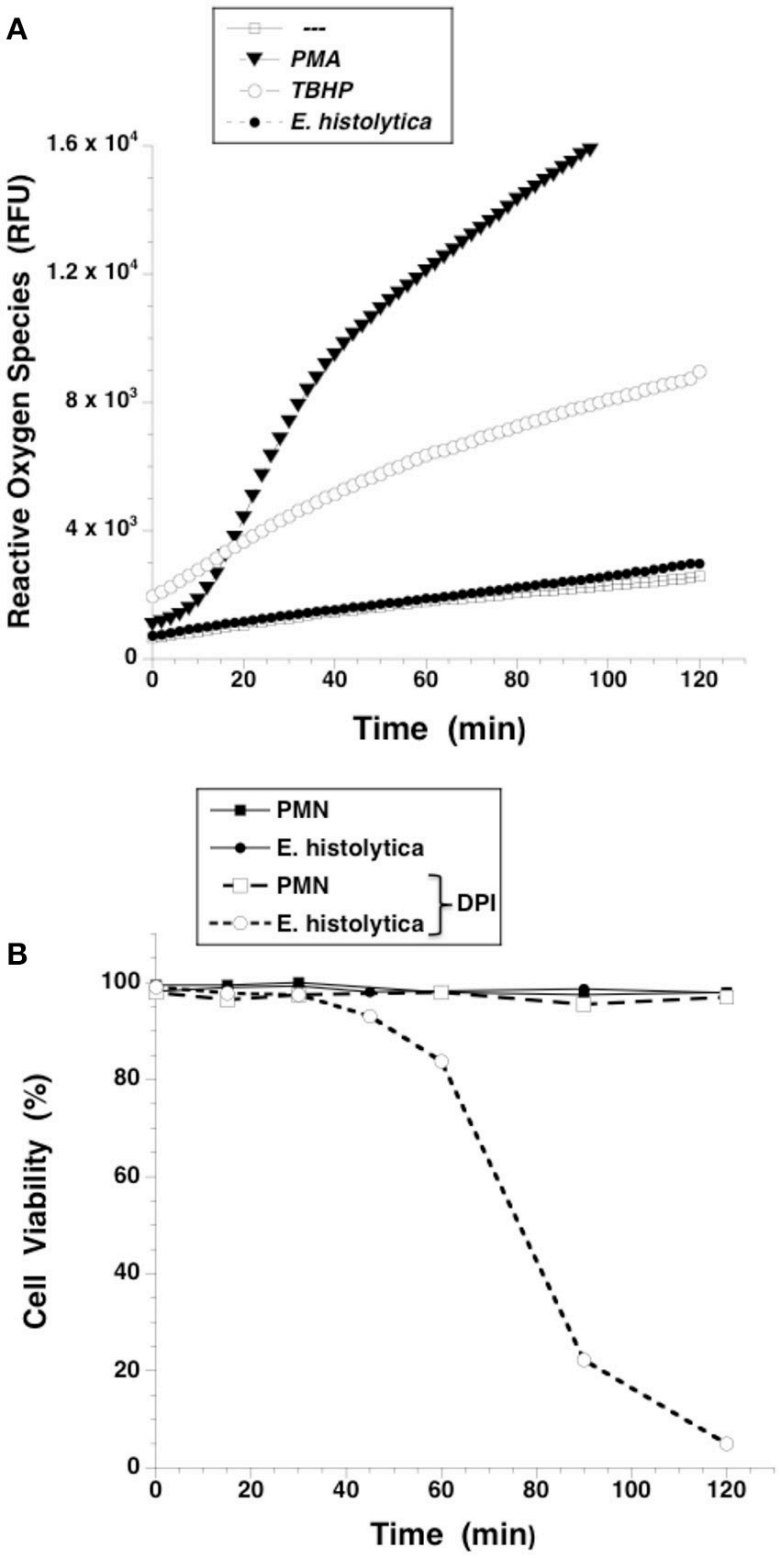
*Entamoeba histolytica* does not induce Reactive Oxygen Species (ROS) formation. **(A)** Human neutrophils (PMN) were previously incubated with the ROS-sensitive fluorescent compound DCFDA (15 μM), and were not stimulated (—), or were stimulated with 20 nM phorbol 12-myristate 13-acetate (PMA), or with 200 μM tert-butyl hydrogen peroxide (TBHP), or with *E. histolytica* trophozoites. Fluorescence was read in a plate-reader for 2 h at 36°C. Data are mean ± SEM of relative fluoresce units (RFU) from three experiments. **(B)** Neutrophils (PMN) or *E. histolytica* trophozoites were treated with solvent alone (black symbols) or with the NADPH-oxidase inhibitor diphenyleneiodonium (DPI) (10 μM) (white symbols). Cell viability was estimated by Trypan Blue exclusion every 15 min.

NETs formed after PMA stimulation require activation of NADPH-oxidase and formation of ROS (Patel et al., 2010; Almyroudis et al., 2013; Björnsdottir et al., 2015). Thus, we explored the involvement of these molecules in *E. histolytica*-induced NETs formation. Neutrophils treated with diphenyleneiodonium (DPI), a NADPH-oxidase inhibitor, were not able to form NETs after PMA stimulation (Figure 8). Similarly, DPI-treated neutrophils did not efficiently form NETs after *E. histolytica* stimulation (Figure 8). However, because inhibition by DPI of *E. histolytica*-induced NETs formation was only to about half (Figure 8), we decided to use apocynin, a different inhibitor of NADPH-oxidase (Kim et al., 2012) and also a ROS scavenger (Heumüller et al., 2008). As expected, apocynin inhibited PMA-induced NETosis, indicating that this mechanism depends on ROS production (Díaz-Godínez et al., 2018). In contrast, apocynin did not decrease amoeba-induced NETs formation, suggesting that this type of NETosis is independent of ROS production by NADPH oxidases (Díaz-Godínez et al., 2018). In order to clarify the different effect of these two NADPH-oxidase inhibitors, we decided to directly assess ROS production after *E. histolytica* stimulation of neutrophils. When neutrophils were treated with PMA or with tert-butyl hydrogen peroxide (TBHP), a positive control for ROS production, ROS were generated in great amounts and could be easily detected with the 2′,7′-dichlorofluorescin diacetate (DCFDA) method (Figure 9A). To our surprise, however, *E. histolytica* stimulation of neutrophils did not induce any ROS production (Figure 9A). Consequently, the effect of DPI did not seem to be related to inhibition of ROS production. Cell viability was then tested in the presence of DPI. Neutrophils remained viable in the presence of DPI for more than 2 h (Figure 9B). In contrast, *E. histolytica* trophozoites began losing viability around 45 min after treatment with DPI. By 90 min, only about 20% amoebas were still viable. Finally, by 2 h most amoebas were not alive (Figure 9B). Therefore, the effect of DPI on NETs production was not due to inhibition of ROS production, but due to a toxic effect on *E. histolytica*.

### *Entamoeba histolytica* did not induce ROS production

**Figure 10:**
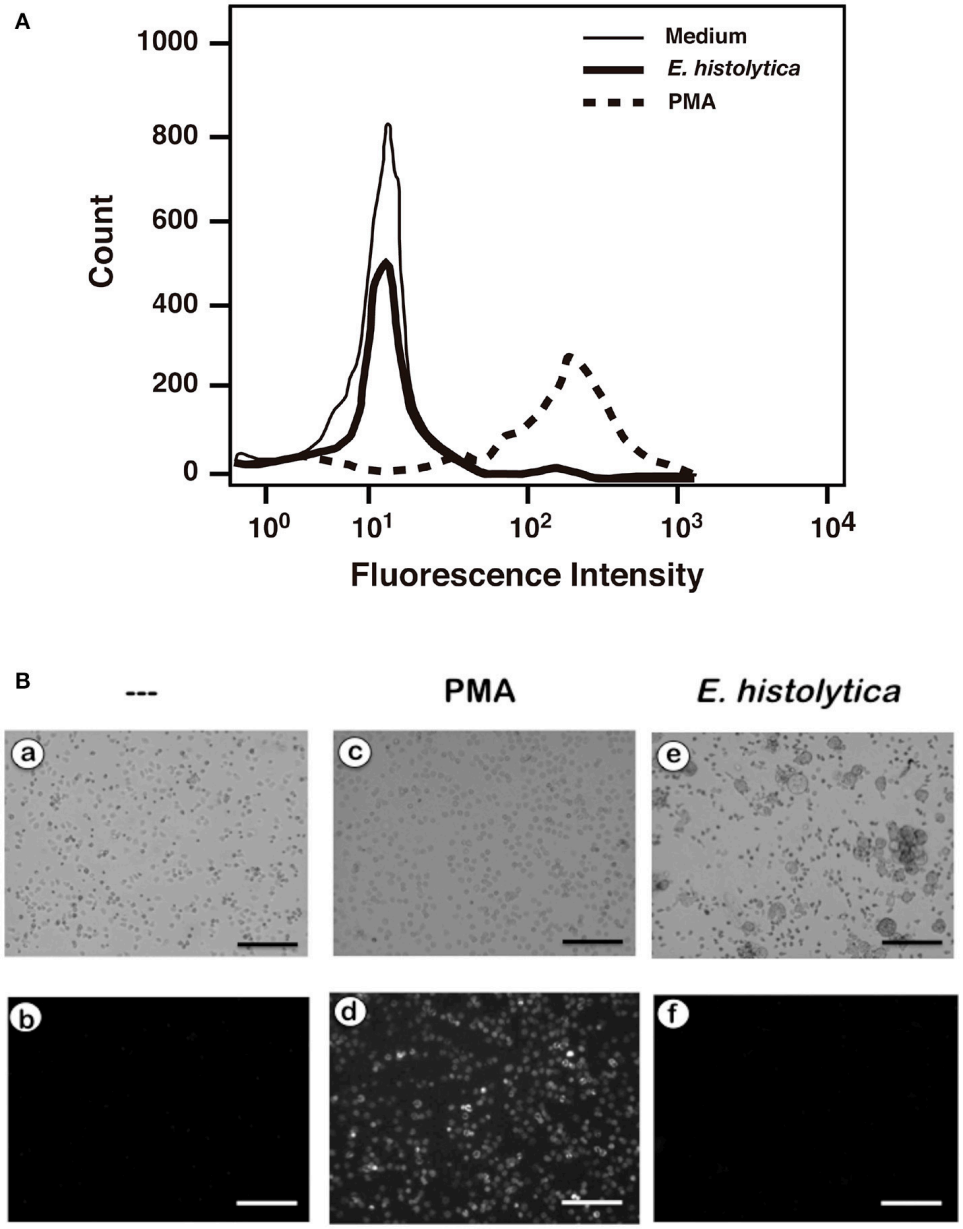
*Entamoeba histolytica* does not induce Reactive Oxygen Species (ROS) formation. Human neutrophils were previously incubated with the ROS-sensitive fluorescent compounds **(A)** dihydrorhodamine 123 (15 μM), or **(B)** dihydroethidium (15 μM). Next, neutrophils were not stimulated (—), or were stimulated with 20 nM phorbol 12-myristate 13-acetate (PMA), or with *E. histolytica* trophozoites. After 1 h, cells were analyzed by flow cytometry **(A)** or by fluorescence microscopy **(B)**. Data are representative of three independent experiments. Bar is 100 μm.

In order to confirm that *E. histolytica* did not induce ROS production, neutrophils were loaded with dihydrorhodamine 123 or with dihydroethidium, and ROS production assessed by flow cytometry and fluorescence microscopy respectively. When neutrophils were treated with PMA, a strong increase in dihydrorhodamine 123 fluorescence could be easily detected by flow cytometry (Figure 10A) indicating the presence of ROS. Similarly, PMA induced ROS could be easily detected by fluorescence microscopy (Figure 10B). In contrast, *E. histolytica* did not cause any rise in fluorescence from these ROS indicators (Figure 10). Thus, clearly *E. histolytica* did not induce any ROS production from neutrophils. These data suggest that amoebas can induce NETs production by a signaling pathway that is independent of ROS.

### *Entamoeba histolytica*-induced NETs formation is dependent on NF-κB

**Figure 11:**
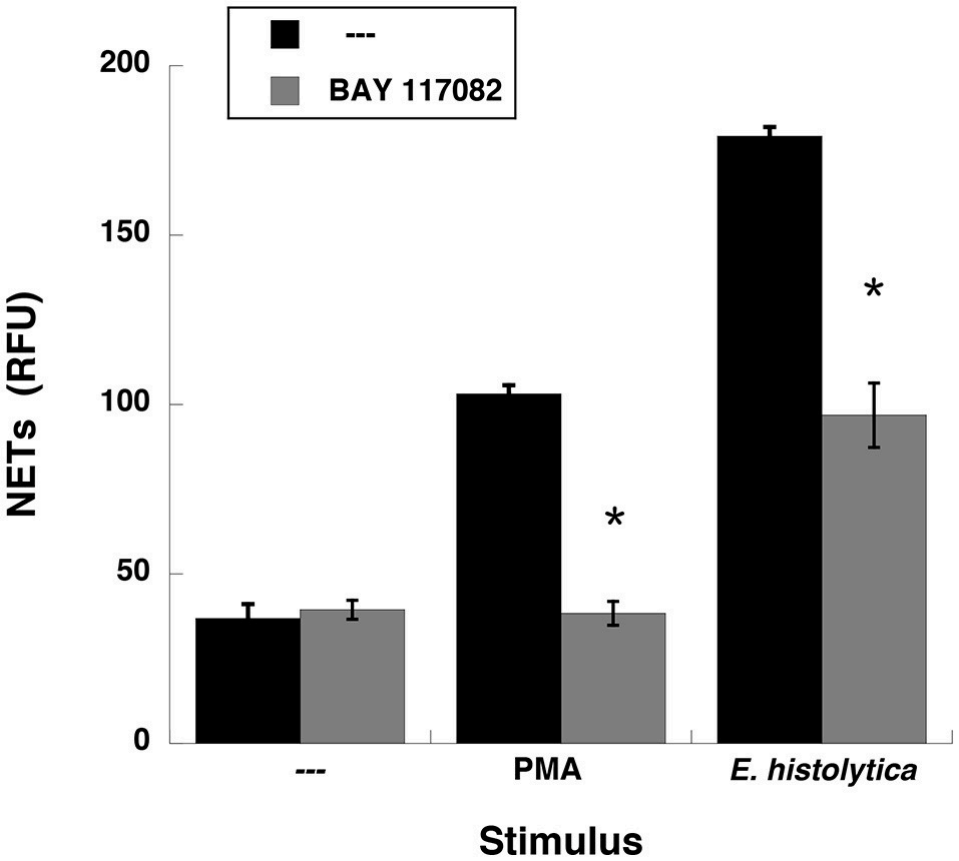
*Entamoeba histolytica-*induced NET formation is dependent on NF-κB. Human neutrophils were not stimulated (—), or were stimulated with 20 nM phorbol 12-myristate 13-acetate (PMA), or with *E. histolytica* trophozoites. Neutrophils were previously treated with solvent alone (—) or with the NF-κB inhibitor BAY 117082 at 5 μM. The relative amount of NETs was estimated from SYTOX® Green fluorescence in relative fluorescent units (RFU) at 4 h after stimulation. Data are mean ± SEM of four experiments. Asterisks denote conditions that are statistically different from control (*p* < 0.03).

**Figure 12:**
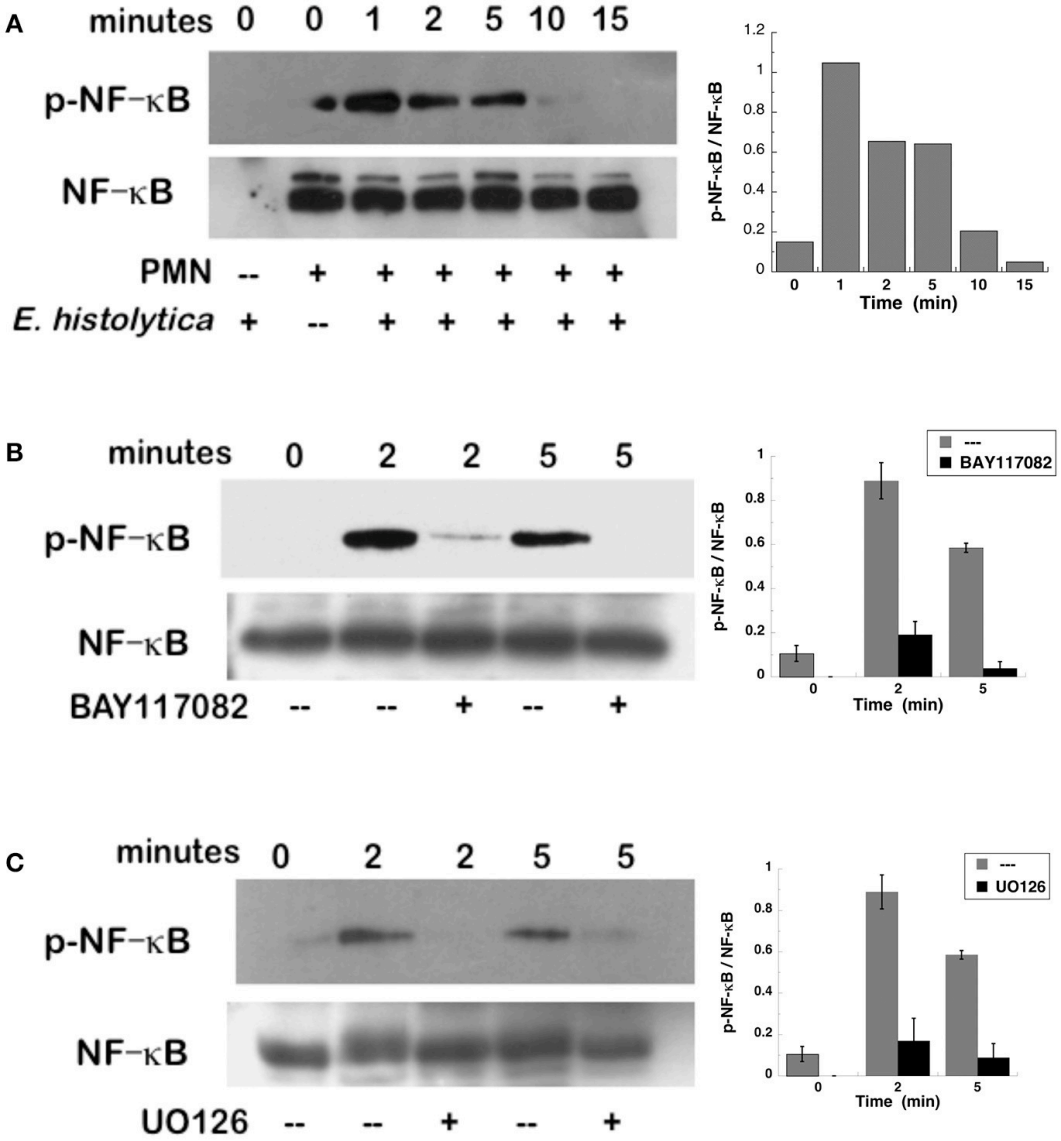
*Entamoeba histolytica* induce activation of NF-κB. Human neutrophils were stimulated with *E. histolytica* trophozoites for various periods of time, and then cell lysates were prepared. Neutrophils were **(A)** left untreated, or previously treated with **(B)** the NF-κB inhibitor BAY117082 (5 μM), or with **(C)** the MEK inhibitor UO126 (75 μM). Proteins were resolved by SDS-PAGE, and then Western blotted for phosphorylated-NF-κB (p-NF-κB) (upper panel) or for total NF-κB (lower panels) to show equal loading of proteins. Plots on the right show densitometric analysis for the ratio of p-NF-κB/NF-κB. Data are mean ± SEM of three experiments.

NETs formation after PMA stimulation requires activation of NF-κB (Lapponi et al., 2013). Thus, we explored the involvement of this molecule in *E. histolytica*-induced NET formation. As previously reported (Alemán et al., 2016a), neutrophils treated with BAY117082, an NF-κB inhibitor, were not able to form NETs after PMA stimulation (Figure 11, Supplementary Figure 4). Similarly, neutrophils treated with BAY117082 did not form NETs efficiently after *E. histolytica* stimulation (Figure 11, Supplementary Figure 4). To confirm that NF-κB was activated after *E. histolytica* stimulation, phosphorylation of NF-κB was detected by Western blotting. *E. histolytica* induced a rapid and transient NF-κB phosphorylation, which reached a maximum at about 1 min after neutrophils and amoebas got in contact (Figure 12A). This phosphorylation then decreased with time and was barely detectable after 10 min. The antibodies used to detect NF-κB and phospho- NF-κB did not recognize any proteins from amoeba cell lysates (Figure 12A). *E. histolytica*-induced NF-κB activation was completely blocked by the NF-κB inhibitor BAY117082 (Figure 12B). In addition, the MEK inhibitor UO126 also blocked *E. histolytica*-induced NF-κB phosphorylation (Figure 12C), indicating that activation of NF-κB is downstream from the ERK signaling pathway. Together these data suggested that amoebas could induce the formation of NETs independently of NADPH-oxidase activation, but with the involvement of NF-κB activation.

### *Entamoeba histolytica*-induced NETs formation is independent on Syk, TAK1, PI3K, p38 MAPK, and β2 integrins

**Figure 13:**
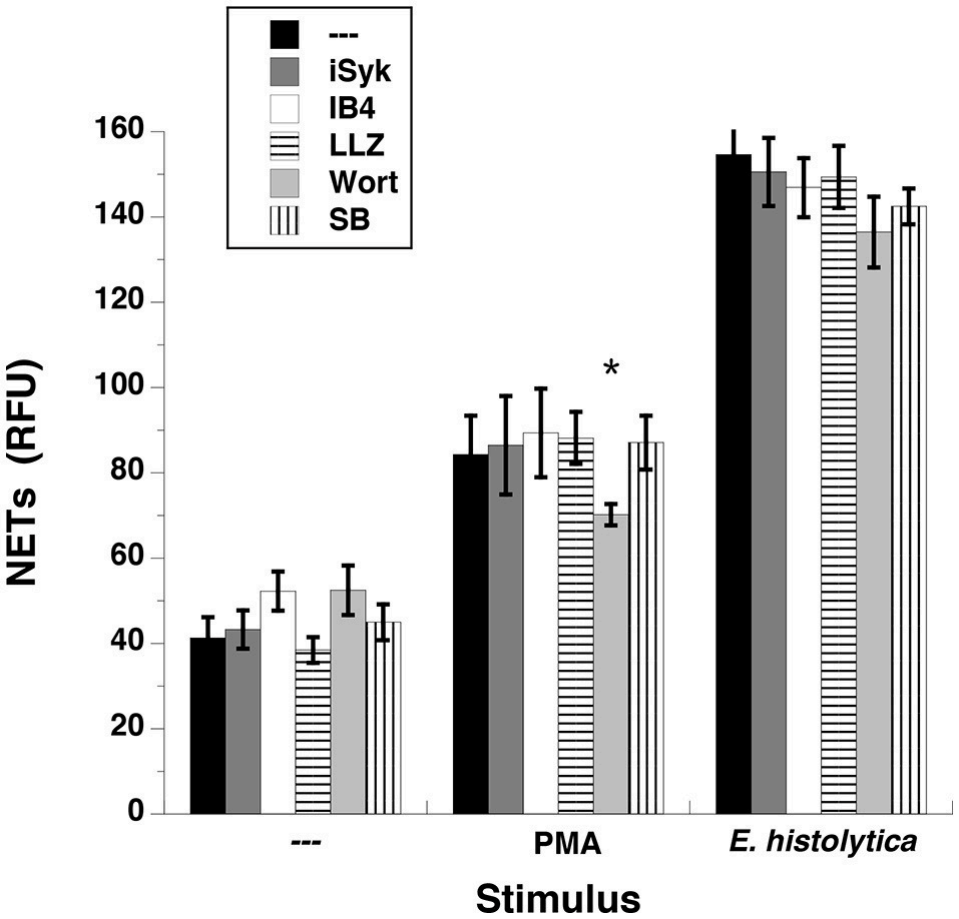
*Entamoeba histolytica-*induced NET formation is independent on Syk, TAK1, β2 integrins, PI3K, and p38 MAPK. Human neutrophils were left untreated (—), stimulated with 20 nM phorbol 12-myristate 13-acetate (PMA), or with *E. histolytica* trophozoites. PMN were previously treated with solvent alone (—) or 1 μM iSyk, a Syk inhibitor, or 10 μg/ml of the blocking monoclonal antibody anti-β2 integrins (IB4), or 10 nM LLZ 1640-2 (LLZ), a TAK1 inhibitor, or 50 nM Wortmannin (Wort), a PI3K inhibitor, or 200 nM SB203580 (SB), a p38 MAPK inhibitor. The relative amount of NETs was estimated from SYTOX® Green fluorescence in relative fluorescent units (RFU) at 4 h after stimulation. Data are mean ± SEM of three experiments. Asterisk denotes a condition that was statistically different from control (*p* < 0.05).

When neutrophils are stimulated through the FcγRIIIb, the kinases Syk and TAK1 are involved in a signaling pathway that leads to NETs formation (Alemán et al., 2016a,b). Similarly some reports suggest that β2 integrins are required for NETs formation (Raftery et al., 2014; Rossaint et al., 2014). Thus, we explored whether these signaling molecules were also involved in *E. histolytica*-induced NETs formation. Neutrophils pretreated with iSyk, a specific Syk inhibitor formed NETs efficiently after both PMA and *E. histolytica* stimulation (Figure 13). Similarly, inhibition of TAK1 with the antibiotic LLZ 1640-2, or inhibition of β2 integrins with the blocking monoclonal antibody IB4 did not have any effect on NETs formation (Figure 13, Supplementary Figure 5). Also, inhibition of phosphatidylinositol 3-kinase (PI3K) with Wortmannin slightly reduced, as previously reported (Alemán et al., 2016a), PMA-induced NETosis, but had no effect on *E. histolytica*-induced NETs formation (Figure 13). Finally, inhibition of p38 MAP kinase with the specific inhibitor SB 203580 did not have any effect on NETs formation (Figure 13, Supplementary Figure 5). Together these data indicate that these signaling molecules are not involved in the signal pathway activated by amoebas to induce NETs formation.

## Discussion

**Figure 14:**
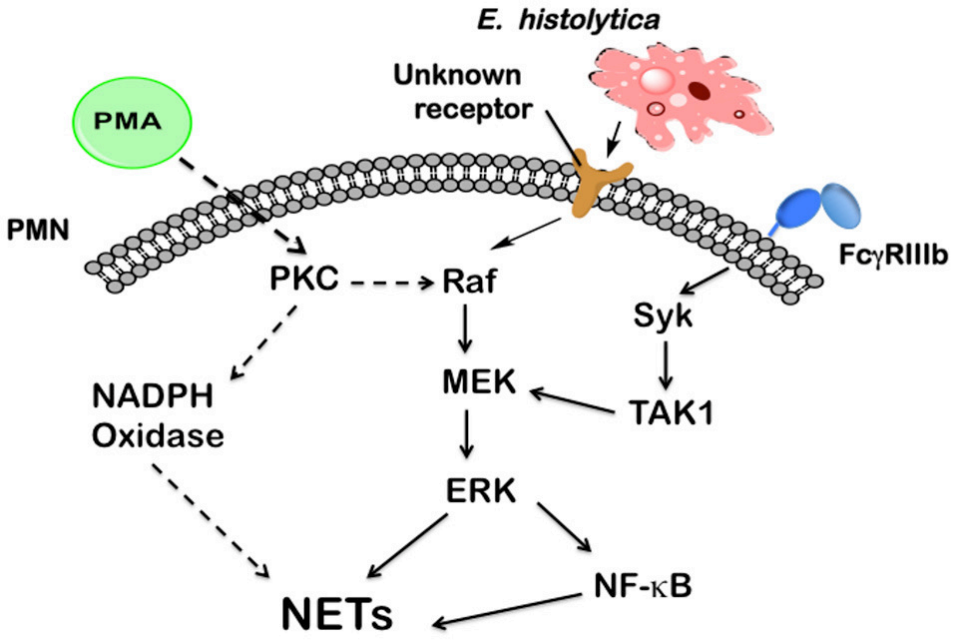
Model for signaling in neutrophils to induce NETosis after *Entamoeba histolytica* engagement. In human neutrophils, phorbol 12-myristate 13-acetate (PMA) can directly activate (dashed arrows) protein kinase C (PKC), which in turn leads to activation of the Raf/MEK/ERK pathway. These kinases finally promote NETs formation. PKC is also required for NADPH-oxidase activation to form reactive oxygen species, which are required for NETs formation after PMA stimulation. In contrast, *E. histolytica* trophozoites are recognized by neutrophils via a, yet unknown, receptor, which connects to the Raf/MEK/ERK pathway. Also the nuclear factor kappa B (NF-κB) is activated to promote NETs formation. The antibody receptor FcγRIIIb also induces NETs formation via spleen tyrosine kinase (Syk) and transforming growth factor-β-activated kinase 1 (TAK1), which connects to MEK (Alemán et al., 2016b). Other signaling molecules (not shown), such as phosphatidylinositol 3-kinase (PI3K), and p38 MAP kinase are not involved in *E. histolytica* signaling to NETs formation.

Neutrophils present several antimicrobial defense mechanisms, including phagocytosis (Rosales and Uribe-Querol, 2017), respiratory burst, degranulation (Kolaczkowska and Kubes, 2013; Mayadas et al., 2014), and the formation of NETs (Yipp et al., 2012). Many pathogens, including virus, bacteria, fungi, and parasites are capable of inducing NETs formation (Papayannopoulos and Zychlinsky, 2009). Although the list of pathogens that induce NETs keeps growing every day, our knowledge about the molecular mechanisms that initiate this neutrophil function is very limited. Recently, we have reported that *E. histolytica* trophozoites were capable of inducing NETosis in human neutrophils (Ávila et al., 2016), but the role of this process in amoebiasis and the molecular mechanisms implicated in NETs formation were not clarified. In this report, we describe for the first time the *E. histolytica*-induced signaling to activate NETs formation. This signaling pathway involves Raf/MEK/ERK, but it is independent of PKC, ROS, Syk, and TAK1 (Figure 14).

Neutrophils, the most abundant leukocytes in blood, are rapidly recruited to sites of infection, where they act as the first line of defense against invading pathogens (Kolaczkowska and Kubes, 2013). Neutrophil activation, through various membrane receptors (Mócsai et al., 2015), is important for initiation of the various defense mechanisms of these cells. NETs are extracellular fibers formed by chromatin covered with histones (Neeli and Radic, 2012) and antimicrobial proteins derived from neutrophil granules (Brinkmann et al., 2004). NETs seem to act as a physical barrier for preventing pathogen disemination (Papayannopoulos and Zychlinsky, 2009). NETs also display antimicrobial activity that is independent of phagocytosis (Urban et al., 2006). Despite the fact that many pathogens, including virus, bacteria, fungi, and parasites (Papayannopoulos and Zychlinsky, 2009) have all been reported to induce NET formation, no particular receptor for PAMPs has been identified on the neutrophil membrane as responsible for inducing this neutrophil response. However, TLRs have been suggested to participate (Yipp et al., 2012). Only two receptors on the human neutrophil have been reported to be genuine activators of NETs release, the IgA receptor FcαR (Aleyd et al., 2014), and the IgG receptor FcγRIIIb (Behnen et al., 2014; Alemán et al., 2016a). In the case of human protozoan parasites, NETs formation has been described to occur in response to *L. amazonensis, L. major, L. chagasi, L. donovani* promastigotes (Guimarães-Costa et al., 2009; Gabriel et al., 2010; Hurrell et al., 2015), *T. gondii* (Abi Abdallah et al., 2012), *T. cruzi* (Sousa-Rocha et al., 2015), and *E. histolytica* (Ávila et al., 2016; Ventura-Juarez et al., 2016). Yet, the mechanism of NETs induction by any of these parasites remains unknown.

Most studies on NETs formation have been conducted with phorbol 12-myristate 13-acetate (PMA) stimulation (Brinkmann et al., 2004; Fuchs et al., 2007). PMA is a direct activator of protein kinase C (PKC), and therefore inhibition of PKC has been shown to block NETs formation (Neeli and Radic, 2013). In agreement with those reports, we found that two different inhibitors of PKC indeed blocked NETs formation after PMA stimulation. In contrast, *E. histolytica*-induced NETs formation was not affected by PKC inhibition. Since PMA directly activates PKC, any possible receptor involved is bypassed. Thus, in the case of amoeba, it seems that the receptor(s) involved can connect with downstream signaling molecules required for NETosis without the need for PKC. This result indicates that PKC is not always necessary for NETs formation. In contrast, the ERK signaling pathway seems to be a common denominator for NETs formation. In the case of PMA-induced NETosis, it was found that PKC leads to activation of the Raf/MEK/ERK pathway (Hakkim et al., 2011). In the case of FcγRIIIb stimulation, we also found that the ERK pathway is important for NETs formation (Alemán et al., 2016a,b). Now, we describe that *E. histolytica* also induces activation of the Raf/MEK/ERK pathway for NETs formation, but independently of PKC (Figure 14). Nevertheless, we could not identify a particular receptor that would recognize amoeba and activate the Raf/MEK/ERK signaling cascade. Several possible receptors on the neutrophil are candidates for amoeba recognition, including some TLRs. Our group continues exploring this line of research.

Whatever the neutrophil receptor for amoeba is, it clearly connects to Raf kinase and activates ERK signaling. At present, there is no information of how Raf can be activated after *E. histolytica* recognition by neutrophils. Since Raf is primarily activated by the small GTPase Ras (Lavoie and Therrien, 2015), it is possible that amoebas trigger Ras activation and in turn Raf signaling. But, Raf can also be activated by several other means, including PKC (Takahashi et al., 1999), other small GTPases (Mishra et al., 2005), and even independently of GTPases (Rouquette-Jazdanian et al., 2012). Receptors for growth factors that are usually receptor tyrosine kinases (RTK) activate Raf via the small GTPases Ras (Lavoie and Therrien, 2015) or Rap1 (Mishra et al., 2005). Yet, some RTK, such as the vascular endothelial growth factor (VEGF) receptor can activate Raf independently of Ras using PKC instead (Takahashi et al., 1999). Similarly, in lymphocytes the T-cell receptor uses Pak1 kinase to activate Raf-1 and MEK independently of Ras or PKC (Rouquette-Jazdanian et al., 2012). Thus, in the case of amoebas, Raf activation could be achieved via either a small GTPase or Pak1 kinase. However, another possibility for Raf activation seems more likely to be involved in amoeba-induced Raf activation. In keratinocytes, stimulation with extracellular calcium resulted in activation of Raf and ERK pathway, without the involvement of Ras (Schmidt et al., 2000). In addition, this Raf activation did not connect to the JNK or p38 pathways. In *E. histolytica*-induced NETs formation, we also found strong calcium mobilization (Figure 5), and ERK but not p38 activation (Figure 13). Therefore, the extracellular calcium flux into neutrophils that are in contact with amoebas may also serve to activate the Raf/MEK/ERK pathway. This possibility is actually being explored in our laboratory.

An important difference between PMA-induced and amoeba-induced as well as FcγRIIIb-induced NETs formation was the time required for NETs release. As previously reported, release of NETs after PMA was detected 3–4 h after stimulation and was dependent on ROS, since the NADPH-oxidase inhibitor DPI abolished NETs release (Brinkmann et al., 2004; Fuchs et al., 2007). In contrast *E. histolytica*-induced NETs release was much rapid and stronger than the one induced by PMA (Figure 3). This response was similar to the rapid, oxidant-independent NETs release described after *Staphylococcus aureus* stimulation of neutrophils (Pilsczek et al., 2010). ROS are required for NETs formation in most cases (Brinkmann et al., 2004, 2010; Fuchs et al., 2007; Parker et al., 2012a), but ROS are not sufficient, since ROS production induced by phagocytosis cannot initiate NETs formation (Branzk and Papayannopoulos, 2013). ROS production was not detected when human neutrophils were mixed with *E. histolytica* trophozoites (Díaz-Godínez et al., 2018) and (Figures 9, 10). Therefore, NETs formed after amoeba recognition by neutrophils seem to be independent of ROS. Because, generation of ROS has been reported during the interaction of neutrophils with *E. histolytica* trophozoites (Sim et al., 2005), our data suggest that the mechanism of NETs formation induced by amoebas is independent of ROS.

Also, it has been previously suggested that interaction of amoebas with neutrophils results in apoptosis (Sim et al., 2005). We did not find evidence for apoptosis of neutrophils interacting with *E. histolytica* trophozoites (Díaz-Godínez et al., 2018). We do not know exactly the reasons for the different results between that initial report and our present results. Apoptosis was evaluated by surface expression of phosphatidylserine and FcγRIIIb (CD16b) by FACS, and by cleavage of caspases in Western blots (Sim et al., 2005). Under conditions similar to those presented in that report (Sim et al., 2005), we did not detect any increase in phosphatidylserine expression (Díaz-Godínez et al., 2018), or any decrease in CD16b expression using two different antibodies anti-CD16 (Figure 6). In addition, no DNA degradation typical of apoptosis could be detected in our cells (Díaz-Godínez et al., 2018). Thus, we think neutrophils do not really undergo apoptosis from interacting with amoebas. Careful reading of the initial report reveals that neutrophils alone kept in culture at 37°C for 1 h entered spontaneously into apoptosis, as suggested by having 27% of neutrophils positive for propidium iodide staining and 29% of neutrophils stained for annexin-V, a marker for phosphatidylserine (Sim et al., 2005). These percentages increased in the presence of *E. histolytica* trophozoites and were interpreted as amoebas inducing apoptosis of neutrophils (Sim et al., 2005). Also, the cleavage of caspases was very high in neutrophils alone (Sim et al., 2005), suggesting that neutrophils were already in apoptosis without interacting with amoebas. In addition, reduction of CD16 expression was used as a marker for apoptotic neutrophils. Although, apoptotic neutrophils show reduced expression of CD16 (Dransfield et al., 1994), the rapid loss of CD16 expression is better associated with the response of neutrophils to inflammatory signals (Moldovan et al., 1999). Moreover, neutrophils respond to inflammatory signals by producing ROS (Mayadas et al., 2014; El-Benna et al., 2016), and we did not find any evidence for ROS production in the presence of amoebas. Hence, it seems that, in the initial report, neutrophils were stimulated by other means, and in consequence some of the cells underwent apoptosis, independently of amoebas.

We believe that a different scenario is taking place when *E. histolytica* trophozoites interact with neutrophils. Upon recognition of trophozoites, only the neutrophils in direct contact with the parasite release DNA fibers that can completely cover the amoeba (Figure 2B). Since, neutrophils cannot phagocytize large cells (Rosales and Uribe-Querol, 2017), they prefer to release NETs in those cases (Urban et al., 2006; Branzk et al., 2014) to prevent the pathogen from escaping. Thus, instead of the amoeba inducing neutrophil apoptosis, it is the neutrophil attacking the trophozoite by undergoing NETosis.

The exact role of NADPH oxidase-dependent ROS for NETs formation remains unclear. When neutrophils were stimulated by *Candida albicans* or by Group B *Streptococcus* (GBS), NETs were formed normally by healthy neutrophils when ROS were eliminated by the ROS scavenger pyrocatechol (Kenny et al., 2017), suggesting a ROS-independent via for NETosis. Yet, neutrophils from chronic granulomatous disease (CGD) patients were not able to form NETs in response to the same stimuli (Kenny et al., 2017), indicating a need for ROS in NETs formation. Possible explanations proposed by the authors are that in the case of GBS there was some residual ROS activity, and in the case of *C. albicans* the fungus itself produces low levels of ROS that the neutrophil can use to activate NETosis (Kenny et al., 2017). In addition, other pathogens, such as *L. amazonensis*, have also been reported to induce NETs in the absence of ROS production (Rochael et al., 2015; DeSouza-Vieira et al., 2016). Together, these studies suggest that PMA absolutely depends on NADPH oxidase derived ROS for NETs formation, while *C. albicans* and GBS can elude this need to some degree, and parasites such as *L. amazonensis* and *E. histolytica* can induce NETosis in complete absence of ROS. In addition to these parasites, various other stimuli can also induce NETosis independently of NADPH oxidase activity, including nicotine, calcium ionophores, uric acid, and immune complexes (Parker et al., 2012b; Arai et al., 2014; Hosseinzadeh et al., 2016; Kraaij et al., 2016). Yet, other sources of ROS such as the mitochondrial respiratory chain or exogenous hydrogen peroxide produced by microorganisms have been considered key for NETosis induced by calcium ionophores (Douda et al., 2015) and by *C. albicans* (Kenny et al., 2017). Therefore, we cannot abandon the possibility that another ROS source, producing amounts that might not be detected with the methodology we used here, play a role in the amoeba-induced NETosis.

Although, the Raf/MEK/ERK pathway has a central role for NETs formation induced by both PMA- and *E. histolytica*, as shown by MEK inhibition blocking NETosis, the role of ERK in NETs formation remains unclear. Previously, it was reported that ERK is required for NADPH-oxidase activation (Hakkim et al., 2011), placing ERK upstream of ROS production. However, it has also been suggested that ROS are downstream of ERK activation (Keshari et al., 2013). Since, as discussed above, ROS are essential for PMA-induced NETosis, but they are not needed for amoeba-induced NETosis, it seems that NADPH-oxidase activation for NET formation, may proceed not only through an ERK pathway, but also independently of ERK activation, depending on the stimulus (Neeli et al., 2009; Kenny et al., 2017). The actual targets downstream of ERK required for NETosis are still unknown.

One possible molecule activated downstream of ERK that has been implicated in PMA-induced NETosis is the nuclear factor kappa B (NF-κB) (Lapponi et al., 2013). Similarly, *E. histolytica*-induced NETosis was blocked when NF-κB activation was prevented (Figures 11, 12). How NF-κB connects to NETs formation is a mystery. No clear function for this transcription factor has been reported. Originally, it was proposed that NF-κB would be required to increase the inflammatory response of neutrophils (Lapponi et al., 2013), but this idea has not been formally tested. In addition, it was reported that upon PMA stimulation, gene transcription does not have any role in NETs formation (Sollberger et al., 2016). Moreover, NF-κB is not always needed for NETosis. In the case of FcγRIIIb-induced NETs formation NF-κB was found not to be involved (Alemán et al., 2016b). Thus, the participation for this transcription factor in NETosis seems to depend on the type of stimuli used, and needs further exploration.

A possible explanation for the role of NF-κB, and other transcription factors, in NETosis has been provided recently. Through transcriptomics analyses of neutrophils, it was shown that the transcriptional activity reflects the degree of DNA decondensation occurring during NETs formation (Khan and Palaniyar, 2017). Interestingly, although both ROS-dependent and ROS-independent NETs formation require transcriptional activity, transcription starts at multiple loci in all chromosomes earlier in the rapid ROS-independent NETosis (induced by calcium ionophore A23187) than in the ROS-dependent NETosis (induced by PMA) (Khan and Palaniyar, 2017). Moreover, extensive citrullination of histones in multiple loci was found only during calcium-mediated NETosis, suggesting that citrullination of histone contributes to the rapid DNA decondensation seen in ROS-independent NETosis (Khan and Palaniyar, 2017). These data are in agreement with our findings that amoeba-induced NETs formation is rapid, requires calcium, is independent of ROS, and presents citrullination of histones. Therefore, the rapid activation of NF-κB (Figure 12) seems a reflection of the earlier transcriptional activity required for the rapid ROS-independent *E. histolytica*-induced NETs formation.

It is now generally recognized that there are several mechanisms of inducing NETs formation (Zawrotniak and Rapala-Kozik, 2013; Kenny et al., 2017; Papayannopoulos, 2018), but the particular signaling pathways involved remain confusing. Other signaling molecules that have been suggested to participate in NETosis initiated by the FcγRIIIb are Syk (Popa-Nita et al., 2009) and transforming growth factor-β-activated kinase 1 (TAK1) (Alemán et al., 2016b); in NETosis initiated by immune complexes is phosphatidylinositol 3-kinase (PI3K) (Behnen et al., 2014); in NETosis initiated by LPS (Neeli et al., 2009) and by yeast (Byrd et al., 2013) are p38 MAP kinase, and β2 integrins. Activation of Syk by PMA is dependent on PKC (Popa-Nita et al., 2009). However, inhibition of Syk with iSyk slightly reduced PMA-induced NETosis (Alemán et al., 2016a) and had no effect on *E. histolytica*-induced NETosis. In the case of FcγRIIIb, iSyk prevented TAK1 phosphorylation and NETs formation (Alemán et al., 2016a). This effect is interpreted as a result of Syk being activated by receptor engagement leading then to TAK1 activation (Figure 14). In the case of *E. histolytica*, the receptor used by neutrophils to recognize amoebas is still unknown. Thus, most likely this putative receptor does not use Syk to deliver a signal for NET formation. Similarly, inhibition of TAK1, PI3-K, or p38 MAP kinase had no effect on *E. histolytica*-induced NETosis (Figure 14). Thus, the putative receptor for *E. histolytica* recognition most likely connects to the Raf/MEK/ERK pathway independently of these signaling molecules (Figure 14). As discussed above, a possible connection for Raf activation might be the extracellular calcium flux.

Blocking β2 integrins with antibodies against both CD11b and CD18 chains prevented NET formation by LPS (Neeli et al., 2009), by β-glucan (Byrd et al., 2013), and by immobilized immune complexes (Behnen et al., 2014). However, integrin ligands are not sufficient to induce NETs formation in isolated neutrophils (Branzk and Papayannopoulos, 2013). Similarly, in our case selective crosslinking of β2 integrins with mAb IB4 also did not induce any NETs formation (Alemán et al., 2016a). Also, the mAb IB4 did not block FcγRIIIb-induced NETs formation (Alemán et al., 2016b). Similarly, blocking β2 integrins with mAb IB4 also did not inhibit *E. histolytica*-induced NETs formation (Figure 13). The involvement of β2 integrins in NET formation might be more related to the adhesion requirement of neutrophils to form NETs (Brinkmann et al., 2010) than to a signaling capacity of the integrin. Therefore, previous reports suggest that β2 integrins cooperate with other receptors to induce NETosis, but our data suggest that β2 integrins do not participate in NETs formation after *E. histolytica* engagement by neutrophils.

In conclusion, to our knowledge, we show for the first time that *E. histolytica* activates a signaling pathway for inducing NETs formation, that involves Raf/MEK/ERK, but it is independent of PKC, ROS, Syk, and TAK1. Hence, amoebas activate neutrophils to release NETs via a different pathway from the pathways activated by PMA or by the IgG receptor FcγRIIIb (Figure 14). Our results also support the idea that various stimuli promote NETs release via different signaling pathways.

## Author contributions

ZF performed most of the experiments and analyzed data. CD-G performed experiments and discussed data. NM performed Western blots. OA performed calcium experiments and discussed data. EU-Q performed live cell imaging and statistical analysis, discussed data, and prepared figures. JC designed the research, analyzed data, and contributed reagents. CR designed the research, performed statistical analysis, prepared figures, organized the references, and wrote the paper.

### Conflict of interest statement

The authors declare that the research was conducted in the absence of any commercial or financial relationships that could be construed as a potential conflict of interest.
