# Supplementary material for: Entamoeba histolytica Induce Signaling via Raf/MEK/ERK for Neutrophil Extracellular Trap (NET) Formation
Source: Front Cell Infect Microbiol. 2018 Jul 4;8:226. doi: 10.3389/fcimb.2018.00226 (PMC6039748; doi:10.3389/fcimb.2018.00226)
Supplement: Supplementary file 1 [file Image_1.pdf]

# *Supplementary Material*

# *Entamoeba histolytica* induce signaling via Raf/MEK/ERK for neutrophil extracellular trap (NET) formation

**Zayda Fonseca[1], César Díaz-Godínez[1], Nancy Mora[1], Omar Rafael Alemán[1], Eileen Uribe-Querol[2], Julio C. Carrero[1]*, Carlos Rosales[1]***

[1] Departamento de Inmunología, Instituto de Investigaciones Biomédicas, Universidad Nacional Autónoma de México, Mexico City, Mexico

[2] División de Estudios de Posgrado e Investigación, Facultad de Odontología, Universidad Nacional Autónoma de México, Mexico City, Mexico

*** Correspondence:**
Dr. Julio César Carrero
carrero@unam.mx
Dr. Carlos Rosales
carosal@unam.mx

## 1 Suplemmentary Figures

## 1.1 Supplementary Figures

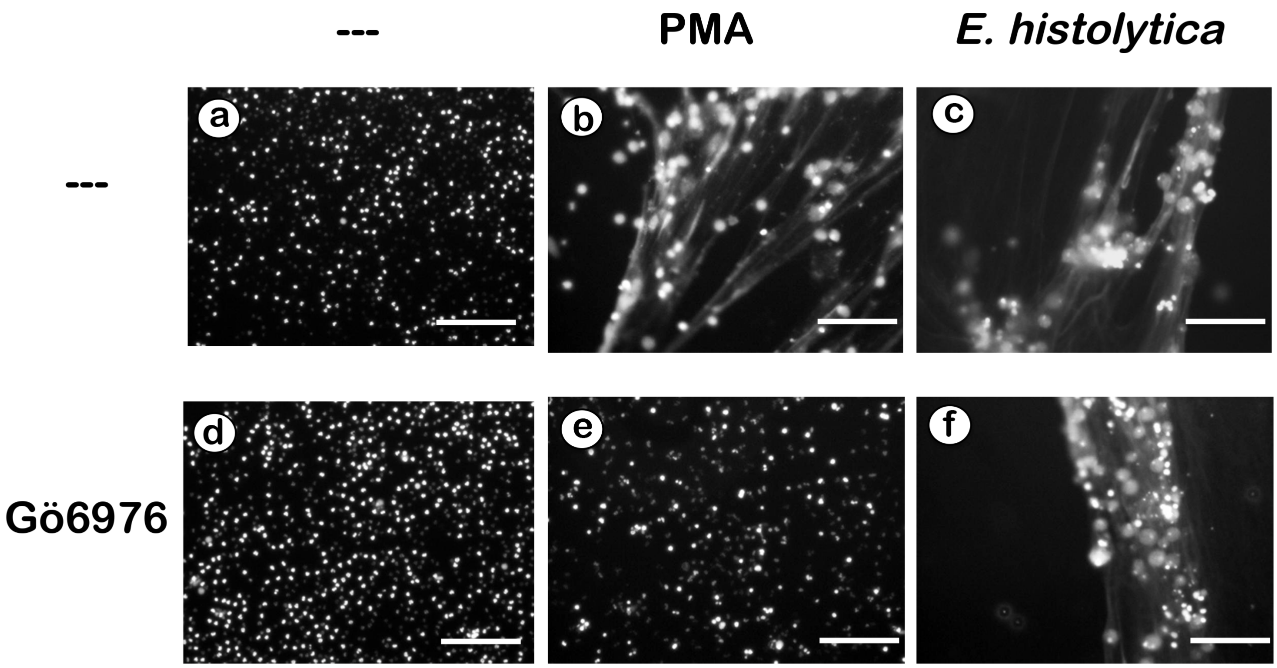


**Supplementary Figure 1.** *Entamoeba histolytica-induced NET formation is independent on PKC.* Human neutrophils were left untreated (---), stimulated with 20 nM phorbol 12-myristate 13-acetate (PMA), or with *E. histolytica* trophozoites. Neutrophils were previously treated with solvent alone (---; panels a, b, c) or with the PKC inhibitor Gö6976 (1 μM) (panels d, e, f). After 4 h, PMN neutrophils fixed and stained for DNA (DAPI). Microphotographs were taken at 200 X magnification and are representative of three experiments. Bar is 100 μm.
