# Supplementary material for: Entamoeba histolytica Induce Signaling via Raf/MEK/ERK for Neutrophil Extracellular Trap (NET) Formation
Source: Front Cell Infect Microbiol. 2018 Jul 4;8:226. doi: 10.3389/fcimb.2018.00226 (PMC6039748; doi:10.3389/fcimb.2018.00226)
Supplement: Supplementary file 4 [file Image_4.pdf]

1.4 Supplementary Figures

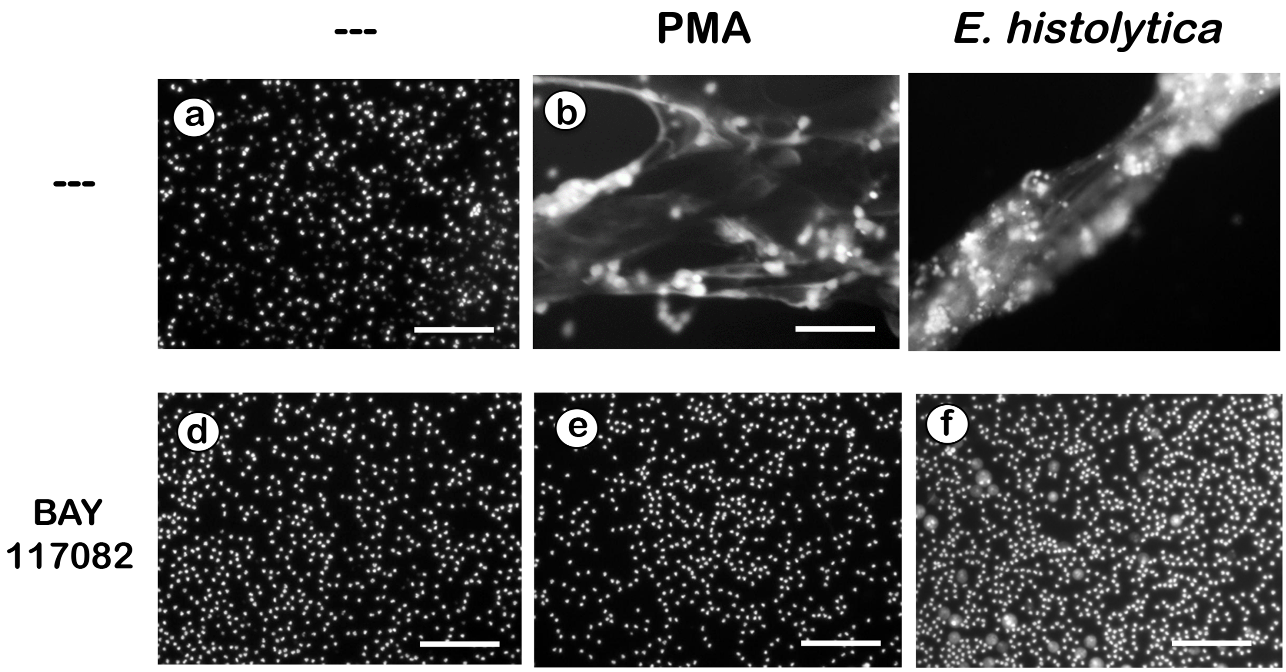

Supplementary Figure 4. *Entamoeba histolytica-induced NET formation is dependent on NF-κB.* Human neutrophils were not stimulated (---), or were stimulated with 20 nM phorbol 12-myristate 13-acetate (PMA), or with *E. histolytica* trophozoites. Neutrophils were previously treated with solvent alone (---; panels a, b, c) or with the NF-κB inhibitor BAY 117082 at 5 μM (panels d, e, f). After 4 h, cells were fixed and stained for DNA (DAPI). Microphotographs were taken at 200 X magnification and are representative of three experiments. Bar is 100 μm.
