# Supplementary material for: Entamoeba histolytica Induce Signaling via Raf/MEK/ERK for Neutrophil Extracellular Trap (NET) Formation
Source: Front Cell Infect Microbiol. 2018 Jul 4;8:226. doi: 10.3389/fcimb.2018.00226 (PMC6039748; doi:10.3389/fcimb.2018.00226)
Supplement: Supplementary file 5 [file Image_5.pdf]

1.5 Supplementary Figures

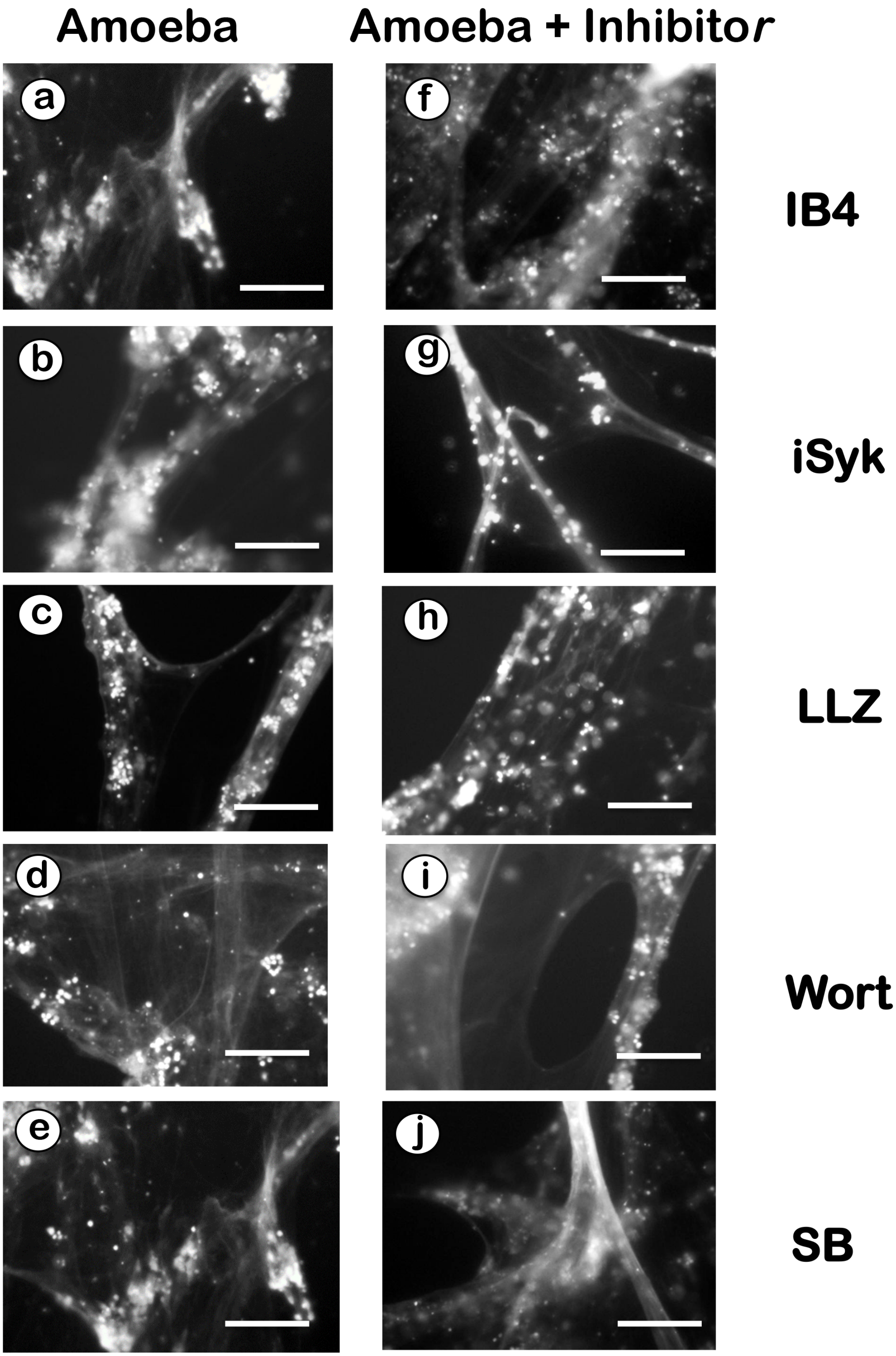

Supplementary Figure 5. *Entamoeba histolytica-induced NET formation is independent on Syk, TAK1, β2 integrins, PI3K, and p38 MAPK*. Human neutrophils were stimulated with *E. histolytica* trophozoites alone (Amoeba) or in the presence of the inhibitors (Amoeba + Inhibitor): 10 μg/ml of the blocking monoclonal antibody anti-β2 integrins (IB4), 1 μM iSyk, a Syk inhibitor, 10 nM LLZ 1640-2 (LLZ), a TAK1 inhibitor, 50 nM Wortmannin (Wort), a PI3K inhibitor, or 200 nM SB203580 (SB), a p38 MAPK inhibitor. After 4 h, cells were fixed and stained for DNA (DAPI). Microphotographs were taken at 200 X magnification and are representative of three experiments. Bar is 100 μm.
